# Validity and timeliness of cancer diagnosis data collected during a prospective cohort study and reported by the English and Welsh cancer registries: a retrospective, comparative analysis

**DOI:** 10.1016/S1470-2045(24)00497-2

**Published:** 2024-11

**Authors:** Ashley Jackson, Pradeep S Virdee, Sharon Tonner, Jason L Oke, Rafael Perera, Kaveh Riahi, Ying Luan, Sara Hiom, Harpal Kumar, Harit Nandani, Kathryn N Kurtzman, Dyfed Huws, Dawn Allan, Stephanie Smits, Sean McPhail, Eileen E Parkes, F D Richard Hobbs, Mark R Middleton, Brian D Nicholson

**Affiliations:** a Department of Oncology, University of Oxford, Oxford, UK; b Nuffield Department of Primary Care Health Sciences, University of Oxford, Oxford, UK; c Abbott Diabetes Care, Witney, UK; d GRAIL Bio UK, London, UK; e GRAIL Menlo Park, CA, USA; f K N Kurtzman Clinical Development, San Francisco, CA, USA; g CDataWise, San Francisco, CA, USA; h Public Health Wales, Welsh Cancer Intelligence and Surveillance Unit, Cardiff, UK; i Swansea University Medical School, Swansea, UK; j National Disease Registration Service, NHS England, Leeds, UK

## Abstract

**Background:**

Cancer places a high burden on society and health-care systems. Cancer research requires high-quality data, which is resource-intensive to obtain. Using administrative datasets such as cancer registries could improve the efficiency of cancer studies if data were valid and timely. We aimed to compare the validity and timeliness of diagnostic cancer data on-site during the SYMPLIFY study to that obtained from the cancer registries of England and Wales.

**Methods:**

Cancer data were collected from 5461 participants across 44 hospital sites during a prospective observational study in England and Wales, SYMPLIFY (ISRCTN10226380). Linked cancer data were obtained from Digital Health and Care Wales (DHCW), the Welsh Cancer Intelligence and Surveillance Unit (WCISU), and the English National Cancer Registration Dataset (NCRD) and Rapid Cancer Registration Dataset (RCRD), regularly between April, 2022, and September, 2023. The primary objectives of the study were to evaluate the validity (via assessment of the proportion of completed data fields and concordance with SYMPLIFY sites), and timeliness of the data in all datasets, for all cancers diagnosed within 9 months of study enrolment. Data fields investigated were cancer site via International Classification of Disease, 10th Revision (ICD-10) code; cancer morphology via International Classification of Diseases for Oncology, 3rd Edition (ICD-O-3) morphology histology code and broad morphological grouping; overall stage; and TNM classification.

**Findings:**

For data collected between April, 2022, and September, 2023, completeness at the last data cut available for each dataset ranged from 84% to 100% for ICD-O-3 morphology, from 43% to 100% for overall stage, and from 74% to 83% for TNM stage. The concordance between SYMPLIFY data and NCRD was 96% (95% CI 92–98) for ICD-10, 60% (53–66) for ICD-O-3 morphology, 83% (78–88) for ICD-O-3 broad morphology groupings, 73% (67–78) for stage, and 51% (44–59) for TNM; and with WCISU was 89% (95% CI 81–94) for ICD-10, 63% (53–73) for ICD-O-3 morphology, 80% (70–87) for ICD-O-3 broad morphology groupings, 83% (74–90) for overall stage, and 49% (38–61) for TNM stage. Concordance between SYMPLIFY and RCRD was 95% (95% CI 92–98) for ICD-10, 67% (60–74) for ICD-O-3 morphology, 85% (79–90) for ICD-O-3 broad morphology groupings, and 73% (65–80) for overall stage; and between SYMPLIFY and DHCW was 96% (91–99) for ICD-10, 74% (64–83) for ICD-O-3 morphology, 84% (75–91) for ICD-O-3 broad morphology groupings, and 87% (74–95) for stage. The SYMPLIFY dataset reached completion at 12 months post-enrolment in November, 2022, compared with 13 months for NCRD in December, 2023. RCRD and DHCW reached completion at 13 months and 15 months post-enrolment, in December, 2022, and February, 2023, respectively.

**Interpretation:**

We report similar completeness of data fields, concordance, and timeliness between on-site and centrally collected cancer outcomes data. Our findings suggest that central registry data can help alleviate the resource burden in clinical trials and improve cancer research. Cancer registries might need additional resources to provide data for registry-based trials at scale.

**Funding:**

GRAIL Bio UK.

## Introduction

Cancer is the second leading cause of death worldwide and it is estimated that half of all people in the UK will be diagnosed with cancer during their lifetime.^1^, ^2^ Although cancer-related mortality continues to decrease, despite the increased incidence, cancer continues to place a substantial burden on health-care systems and affected individuals.^3^ Cancer research is a costly endeavour and approximately a third of the total cost of cancer clinical trials is spent on non-clinical activities, including completing case report forms, data cleaning, and data management.^4^ Registry-based trials can improve the efficiency of clinical cancer research by leveraging population-based cancer registration data as an alternative to data collection by research staff at study sites.^5^ Cancer registries obtain information from a variety of sources and, in contrast with clinical trials, are not confined to standard follow-up periods, which might allow for a more comprehensive follow-up. A modelling study in cardiology found the use of central registry data more cost-effective 98·6% of the time, with savings ranging from $4300 to $600 000, depending on the study design.^6^ Cost savings are linked to a reduced need for data collection and monitoring, staff training, and follow-up visits.^7^, ^8^, ^9^ Registry-based trials can also improve external validity, enable rapid assessment of eligibility, ensure diversity in recruitment, and facilitate follow-up.^5^, ^10^, ^11^, ^12^

**Research in context**

**Evidence before this study**

We searched PubMed for articles in English from database inception to Jun 18, 2024, for “cancer” [title and abstract] AND “registry data” [any field] AND “comparison” [any field] (and related terms). We identified numerous studies conducted in Europe and North America that evaluated the data quality of central cancer databases. The studies by Parkin and Bray outline the important data quality characteristics for cancer registries: comparability, validity, timeliness, and completeness. We identified a study of English bowel cancer registrations between 1996 and 2004, which demonstrated a stage completion of 60% for bowel cancer registration. However, this study was conducted before the launch of the National Cancer Registration and Analysis Service (NCRAS) in 2013. The cluster randomised CAP trial of prostate-specific antigen testing for prostate cancer compared the completeness and concordance of prostate cancer data collected during their study to that of the National Cancer Registration Dataset. However, the CAP study was conducted only for prostate cancer and was conducted using cancers diagnosed between 2002 and 2015, most of which pre-dated the development of NCRAS.

**Added value of this study**

To our knowledge, this is the first study to comprehensively compare cancer outcomes data collected on-site by research staff during a prospective study with centrally held registry cancer data in England and Wales. This study demonstrates that centrally held cancer data in both jurisdictions had similar completeness, concordance, and timeliness to cancer data collected on-site.

**Implications of all the available evidence**

We demonstrate that timely data acquisition that is of comparable quality to site-collected study data is possible from national cancer registries in England and Wales if appropriate resourcing is in place. Our results demonstrate moderate concordance between local and central data for broader groupings of cancer site, morphology, and TNM staging, but less so for more granular comparisons. These findings help support researchers in determining whether using centrally collected data is feasible for their study design and desired outcomes. The use of centrally collected cancer data in clinical studies could help to reduce the resource burden associated with on-site follow-up of participants. However, further work is needed to determine if these findings transfer to other cancer data such as treatment and survival data.

The validity of cancer data refers to the proportion of cases with a given attribute and are accurately recorded in the registry data.^13^ Thus, validity depends on the completeness and accuracy of data fields. Ensuring cancer registry data is timely and valid is essential for research; however, obtaining complete and accurate data is often at odds with the timeliness of data acquisition.^13^ The gold-standard national cancer registry for England is the National Health Service (NHS) National Cancer Registration Dataset (NCRD), which uses quality control and assurance checks to maintain validated cancer registration information.^14^ The Rapid Cancer Registration Dataset (RCRD) is also available, enabling near real-time analysis of cancer data in England from 2018 onwards.^15^ The Welsh Cancer Intelligence and Surveillance Unit (WCISU) serves as the national cancer registry in Wales, and Digital Health and Care Wales (DHCW), while not a cancer registry, can provide administrative real-time and longitudinal data including cancer diagnoses.

We aimed to compare cancer data obtained on-site during a prospective cohort study in England and Wales (SYMPLIFY) with data from English and Welsh national cancer registries.^16^ In comparing on-site and registry cancer data, we aimed to assess the feasibility of using these central datasets by examining data validity (via the proportion of completed data fields and concordance of data fields with SYMPLIFY sites) and timeliness.

## Methods

### Study design and participants

The observational SYMPLIFY cohort study (ISRCTN10226380) evaluated a multi-cancer early detection test in symptomatic patients. Eligibility criteria, nature of consent, and ethics approval have been described in detail in the SYMPLIFY study.^16^ 6238 participants were recruited to the SYMPLIFY study between July 7 and Nov 30, 2021, and 5461 participants across 44 hospital sites with a diagnostic resolution reached were included in the final analysis.^16^ These participants were referred by their primary care physician to a rapid diagnostic centre or a gynaecological, lung, upper or lower gastrointestinal 2-week-wait pathway for the rapid investigation of cancer. An exploratory objective of SYMPLIFY was to evaluate the completeness and quality of patient data collected from central databases by evaluating the proportion of completed data fields, according to locally and centrally sourced inputs.

Recruiting hospital sites reviewed the local hospital records for all participants at 3 months post-enrolment, entering data regarding cancer diagnoses into electronic case report forms hosted in a secure online RedCap database. Those that were unresolved at 3 months underwent another review at 9 months. Data cuts were retrieved monthly from SYMPLIFY sites from April, 2022, to January, 2023. Data extraction was carried out by research staff employed at each recruiting site. Complex clinical queries were resolved by the clinical principal investigator at each site. Written informed consent was obtained from participants for the SYMPLIFY team to retrieve data held in central national databases. The protocol was approved by the National Research Ethics Service (21/LO/0456–London Central) and complied with UK regulations.

### Procedures

**Figure 1:**
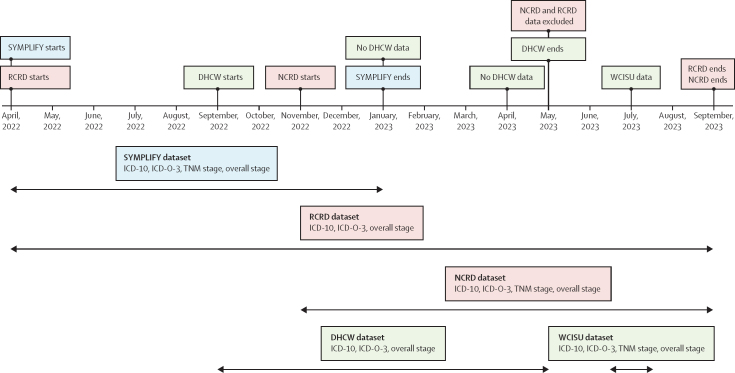
Timeline of the data cuts and data fields available for each dataset DHCW=Digital Health and Care Wales. ICD-10=International Classification of Diseases, 10th Revision. ICD-O-3=International Classification of Diseases for Oncology, 3rd Edition. NCRD=National Cancer Registration Dataset. RCRD=Rapid Cancer Registration Dataset. WCISU=Welsh Cancer Intelligence and Surveillance Unit.

Cancer registry data were collected from WCISU for Wales and from NCRD for England. Rapid cancer data were collected from DHCW in Wales and RCRD in England. Monthly data cuts from RCRD were accessed from April, 2022, to September, 2023; from NCRD from November, 2022, to September, 2023; from DHCW from September, 2022, to May, 2023; and there was a single data cut from WCISU in July, 2023 (figure 1). Due to a data issue at one of the England hospital sites in the May, 2023 data cut, these data have been excluded from the analysis. Each data cut included the most current data available for cancers diagnosed within 9 months of study enrolment. All 5461 patients enrolled in the study were followed up in the cancer registry, regardless of diagnostic status, according to the SYMPLIFY dataset in NCRD, RCRD, and DHCW. Due to resource limitations and later engagement, WCISU was only able to provide data on patients with confirmed cases of cancer, according to DHCW. Early engagement with NHS England in advance of the SYMPLIFY study allowed for timely data acquisition from RCRD and NCRD.

We assessed four data fields among cancers diagnosed within 9 months of enrolment: cancer site using the International Classification of Diseases, 10th Revision (ICD-10) code; cancer morphology using the International Classification of Diseases for Oncology, 3rd Edition (ICD-O-3) morphology code; stage at diagnosis; and TNM classification at diagnosis. Cancer data were evaluated on a cancer level rather than a patient level to account for those who had more than one cancer diagnosed throughout the study period.

ICD-10 codes indicate specific cancer sites and can be grouped to reflect broader site groupings. For example, C18.0–C18.8 all indicate a colon cancer but vary in where the cancer is located along the bowel.^17^ Broad groupings of ICD-10 codes by related cancer sites were used to compare concordance between datasets. If a code was listed that did not correspond with an ICD-10 code, the cancer site was listed as “Unknown Code”. Following the inclusion criteria of SYMPLIFY, only ICD-10 codes starting with “C” or D45–D46.9, D47.3, or D47.4 were included in each dataset, while non-melanoma skin cancers (C44) were excluded.^17^

ICD-O-3 codes include a topographical code, indicating where a given cancer has arisen in the body, a histological code, indicating the cancer morphology, and a behaviour code, indicating the nature of the tumour.^18^ The topographical code uses similar coding to ICD-10, so only the histological code was considered. Only cancers in each dataset with an ICD-O-3 behaviour code of 3, corresponding to a malignant neoplasm, were included in the study. Both the 4-digit histological codes and broader ICD-O-3 groupings were evaluated (appendix pp 31–52).^18^

Stage was defined as the overall stage (I–IV) at diagnosis based on the best available evidence. Stage 0 cancers, corresponding to carcinoma in situ, were excluded from the analysis of each dataset. Some datasets included sub-staging information (eg, stage IIa, IIb) while others employed broad categories (ie, I–IV). To enable comparison between datasets, only the broad stage was considered. The SYMPLIFY dataset used one of the following staging systems to decipher overall stage: International Union Against Cancer (UICC) 8th edition, European Neuroendocrine Tumour Society (ENETS), International Federation of Gynaecology and Obstetrics (FIGO), Ann Arbor, Binet, or other. WCISU used UICC 8th edition, FIGO, and Ann Arbor staging systems, while NCRD used UICC 8th edition, Ann Arbor, ENETS, FIGO, International Staging System, and Binet staging systems.

TNM staging was evaluated as a single complete variable comprised of all three categories (tumour, node, and metastasis). TNM data were recorded across datasets including both numeric and letter coding, therefore full TNM data were considered for concordance purposes. The systems used to record TNM stage were the same as outlined for SYMPLIFY. The TNM staging variables used for NCRD and WCISU utilised UICC 8th edition staging only. Although other variables exist in the NCRD and WCISU datasets to incorporate other non-TNM staging systems (eg, FIGO), these were not included in the study. TNM data were unavailable to us from the DHCW and RCRD datasets and, therefore, these datasets were excluded from TNM comparison.

### Outcomes

The primary study objective was a descriptive analysis comparing the completeness of variables, concordance, and timeliness of diagnostic cancer data collected on-site during the SYMPLIFY study to that of the administrative and cancer registry datasets in England and Wales. Secondary objectives included a descriptive analysis of the discordant cancer site, ICD-O-3 morphology, and overall stage cases between SYMPLIFY and the respective registry datasets.

### Data analysis

Validity of cancer data in central registries was assessed via the proportion of completed data fields and the concordance of those data fields with the SYMPLIFY dataset.

The completeness of data fields was reported as the number and proportion of cancers, including exact binomial confidence intervals, with available data among all cancers in that dataset per data cut. Given that inclusion in the study required cancers to have a reported ICD-10 code that satisfied predefined inclusion criteria, and therefore every cancer in the study had an ICD-10 code complete, ICD-10 completeness was assured and therefore not reported. TNM staging data were considered complete if there was a non “N/A” listing for each of T, N, and M categories. For TNM staging, a listing of TX, NX, or MX was considered complete. For the remaining datapoints a blank field was considered incomplete and “Uncertain” was considered complete. The reason for classifying “Uncertain” as complete was to not underestimate completeness in cases where there was no staging system available or in cases in which the patient was unable to undergo further staging information. More information on the proportion of stage cases labelled as “Uncertain” in our analysis of discordant stage cases is available in the appendix (pp 26–27).

The concordance of data fields was assessed for cancers that were recorded for the same patient in the SYMPLIFY and registry datasets. Concordance was defined as the same data field value reported in both datasets. The number and proportion of concordant cancers with exact binomial confidence intervals were reported. Only cancers that had the given data field completed in SYMPLIFY, and the respective national registry, were considered in the concordance analyses, so as not to bias the results and underestimate true concordance due to missing data. In cases for which patients had multiple cancers reported in one or more of the datasets, these were handled on a case-by-case basis to determine which cancers were most appropriate for comparison. Concordance comparisons were still carried out for other variables when cancer site was discordant to account for potential issues such as inputting errors. When available, data cuts from SYMPLIFY and central databases from equivalent time points were compared. Data cuts from the central databases beyond January, 2023 were compared with the final January, 2023 SYMPLIFY data cut. For ICD-10 codes, concordance was based on the broad ICD-10 groupings. For ICD-O-3 morphology, concordance was determined on the basis of the 4-digit morphology code and the broader morphology groupings.^18^ For overall stage, concordance was based on broad stages I–IV. TNM concordance was based on an overall complete match of TNM staging. Additional post-hoc sub-analyses were conducted comparing concordance between site and registry datasets for individual T category, N category, and M category and are available in the appendix (p 17). For both overall stage and TNM stage, concordance was assessed regardless of whether the same staging system was used in the SYMPLIFY and respective registry datasets. Final concordance refers to the concordance between SYMPLIFY and the registry datasets at the last respective timepoints available for comparison.

Timeliness was evaluated by comparing monthly data cuts to the final data cut for each data source. With only one WCISU data cut available, timeliness could not be investigated for the WCISU dataset. The timeliness of completeness was expressed as the number and percentage of data recorded at each timepoint compared with the final timepoint. The timeliness of concordance was reported as the number and proportion of cancers at each timepoint concordant with the final timepoint. Full concordance refers to the timeliness of concordance and is the point at which the concordance with the last data cut available for the given dataset first reaches 100%.

Additional exploratory analyses were conducted amongst discordant cases among ICD-10 codes, ICD-O-3 morphology groupings, and stage. To investigate discordant cases, the differences in ICD-10 codes and ICD-O-3 broad morphology groupings between the final datasets were summarised. For stage, we compared discordant cases at each time point to determine whether the stage listing was higher in the registry or SYMPLIFY. Additional post-hoc sub-analyses were conducted whereby we investigated the timeliness of cancers that were reported in the national registries but not recorded in the SYMPLIFY dataset to determine what percentage of these cancers fell within the 3-month post-enrolment reporting period. All analyses were completed in Stata (version 17·0) and figures were prepared in GraphPad Prism (version 9.4.1.).

### Role of the funding source

The funding source participated in the study design and in writing of the report, but had no role in data collection, data analysis, or data interpretation.

## Results

The median age at registration of the 5461 participants included in SYMPLIFY who were recruited between July 7 and Nov 30, 2021, was 61·9 years (IQR 53·4–73·0); 3609 (66·1%) of the participants were female and 1852 (33·9%) were male, 4370 (80·0%) were recruited in England and 1091 (20·0%) were recruited in Wales (data not shown).^16^ The timings of the data cuts from SYMPLIFY, NCRD, RCRD, WCISU, and DHCW, and the data fields available from each dataset are shown in figure 1. By the final data cut for each dataset, there were 259 cancers recorded within 9 months of enrolment in 250 participants in SYMPLIFY England, 121 cancers (118 participants) in SYMPLIFY Wales, 226 cancers (221 participants) in RCRD, 291 cancers (276 participants) in NCRD, 122 cancers (118 participants) in DHCW, and 112 cancers (108 participants) in WCISU (table; appendix p 3).

**Table:** Cancers recorded in each dataset at each timepoint

	**SYMPLIFY**		**RCRD (England)**	**NCRD (England)**	**DHCW (Wales)**	**WCISU (Wales)**
	England	Wales				
April, 2022	166 (163, 3·7%)	87 (84, 7·7%)	198 (195, 4·5%)	··	··	··

May, 2022	186 (183, 4·2%)	98 (94, 8·6%)	194 (191, 4·4%)	··	··	··

June, 2022	189 (186, 4·3%)	101 (96, 8·8%)	200 (197, 4·5%)	··	··	··

July, 2022	191 (186, 4·3%)	102 (97, 8·9%)	211 (208, 4·8%)	··	··	··

August, 2022	189 (184, 4·2%)	100 (95, 8·7%)	222 (218, 5·0%)	··	··	··

September, 2022	202 (197, 4·5%)	100 (95, 8·7%)	223 (219, 5·0%)	··	121 (117, 10·7%)	··

October, 2022	244 (246, 5·6%)	116 (111, 10·2%)	223 (219, 5·0%)	··	121 (117, 10·7%)	··

November, 2022	259 (250, 5·7%)	121 (118, 10·8%)	224 (220, 5·0%)	289 (275, 6·3%)	121 (117, 10·7%)	··

December, 2022	259 (250, 5·7%)	121 (118, 10·8%)	226 (222, 5·1%)	292 (278, 6·4%)	121 (117, 10·7%)	··

January, 2023	259 (250, 5·7%)	121 (118, 10·8%)	225 (221, 5·1%)	291 (277, 6·3%)	··	··

February, 2023	··	··	225 (221, 5·1%)	292 (277, 6·3%)	122 (118, 10·8%)	··

March, 2023	··	··	225 (221, 5·1%)	292 (277, 6·3%)	122 (118, 10·8%)	··

April, 2023	··	··	226 (221, 5·1%)	292 (277, 6·3%)	··	··

May, 2023	··	··	··	··	122 (118, 10·8%)	··

June, 2023	··	··	225 (220, 5·0%)	292 (277, 6·3%)	··	··

July, 2023	··	··	225 (220, 5·0%)	282 (277, 6·3%)	··	112 (108, 9·9%)

August, 2023	··	··	226 (221, 5·1%)	291 (276, 6·3%)	··	··

September, 2023	··	··	226 (221, 5·1%)	291 (276, 6·3%)	··	··


Data are number of cancers in dataset (n patients with cancer, proportion of total number of patients). Proportions are derived from the 4370 participants recruited in England for RCRD, NCRD, and SYMPLIFY England datasets, and 1091 participants recruited in Wales for DHCW, WCISU, and SYMPLIFY Wales. DHCW=Digital Health and Care Wales. NCRD=National Cancer Registration Dataset. RCRD=Rapid Cancer Registration Dataset. WCISU=Welsh Cancer Intelligence and Surveillance Unit.

**Figure 2:**
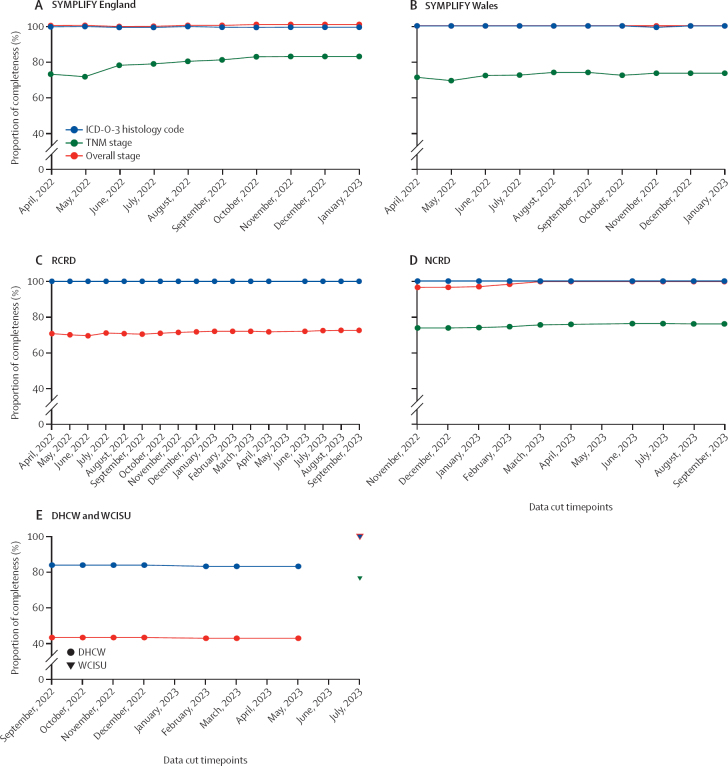
Completeness of data fields for SYMPLIFY England (A), SYMPLIFY Wales (B), RCRD (C), NCRD (D), and DHCW and WCISU (E) datasets ICD-O-3 and stage completion were all approximately 100% in SYMPLIFY England, SYMPLIFY Wales, NCRD, and WCISU, leading to overlapping lines. TNM stage data were not collected for RCRD and DHCW. DHCW=Digital Health and Care Wales. ICD-O-3=International Classification of Diseases for Oncology, 3rd Edition. NCRD=National Cancer Registration Dataset. RCRD=Rapid Cancer Registration Dataset. WCISU=Welsh Cancer Intelligence and Surveillance Unit.

At the final timepoint available for each dataset, the number of cancers with completed morphology data for ICD-O-3 was 255 (98% [95% CI 96–100]) of 259 for SYMPLIFY England, 121 (100% [97–100]) of 121 for SYMPLIFY Wales, 226 (100% [98–100]) of 226 for RCRD, 291 (100% [99–100]) of 291 for NCRD, 102 (84% [76–90]) of 122 for DHCW, and 112 (100% [97–100]) of 112 for WCISU, while overall stage completion was 259 (100% [99–100]) of 259 for SYMPLIFY England, 121 (100% [97–100]) of 121 for SYMPLIFY Wales, 164 (73% [66–78]) of 226 for RCRD, 290 (100% [98–100]) of 291 for NCRD, 53 (43% [34–53]) of 122 for DHCW, and 112 (100% [97–100]) of 112 for WCISU (figure 2, appendix pp 8–10). There was an increase in stage completion in NCRD (100%) compared with RCRD (73%) and similarly, in WCISU (100%) compared with DHCW (43%). TNM completion was 214 (83% [95% CI 77–87]) of 259 for SYMPLIFY England, 89 (74% [65–81]) of 121 for SYMPLIFY Wales, 222 (76% [71–81]) of 291 for NCRD, and 86 (77% [68–84]) of 112 for WCISU. The completeness of data fields in RCRD, NCRD, and DHCW datasets was consistent over time (figure 2).

For SYMPLIFY England participants, 199 cancers (77%) of 259 cancers were reported in RCRD and 236 (91%) of 259 in NCRD (appendix p 11). For SYMPLIFY Wales, 106 (88%) of 121 were reported in DHCW and 98 (81%) of 121 were reported in WCISU (appendix p 11). Investigations of cancers reported in SYMPLIFY England but not NCRD, and those reported in SYMPLIFY Wales but not WCISU, are available in the appendix (pp 24–25).

**Figure 3:**
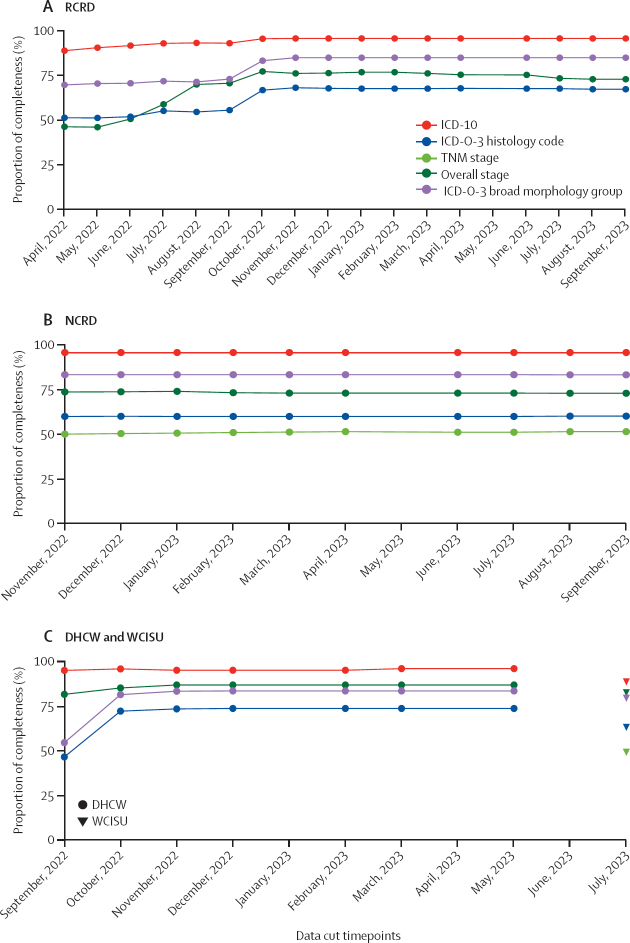
Concordance between data fields in SYMPLIFY and RCRD (A), NCRD (B), and DHCW and WCISU (C) ICD-O-3 histology code is an exact match on the 4-digit histology code. These codes were grouped together into broader morphology classifications to further assess concordance due to the overlap in the histology codes (appendix pp 31–52). TNM stage data were not collected for RCRD and DHCW. DHCW=Digital Health and Care Wales. ICD-10=International Classification of Diseases, 10th Revision. ICD-O-3=International Classification of Diseases for Oncology, 3rd Edition. NCRD=National Cancer Registration Dataset. RCRD=Rapid Cancer Registration Dataset. TNM=Tumour, Nodes, and Metastasis classification. WCISU=Welsh Cancer Intelligence and Surveillance Unit.

Final concordance between SYMPLIFY England and RCRD was 190 (95% [95% CI 92–98]) of 199 for ICD-10, 133 (67% [60–74]) of 198 for ICD-O-3 morphology histological code, 168 (85% [79–90]) of 198 for ICD-O-3 broad morphology groupings, and 107 (73% [65–80]) of 147 for overall stage. Final concordance between SYMPLIFY England and NCRD was 226 (96% [95% CI 92–98]) of 236 for ICD-10, 139 (60% [53–66]) of 232 for ICD-O-3 morphology histological code, 193 (83% [78–88]) of 232 for ICD-O-3 broad morphology groupings, 90 (51% [44–59]) of 176 for TNM, and 171 (73% [67–78]) of 235 for overall stage (figure 3A and B; appendix pp 12–16). Concordance between SYMPLIFY and NCRD was consistent over time while RCRD demonstrated an increase in concordance for all data fields over time (figure 3). TNM concordance was low between SYMPLIFY and NCRD but was higher for individual T, N, and M categories (appendix p 17).

Final concordance between SYMPLIFY Wales and DHCW was 102 (96 [95% CI 91–99]) of 106 for ICD-10, 68 (74% [64–83]) of 92 for ICD-O-3 morphology histological code, 77 (84% [75–91]) of 92 for ICD-O-3 broad morphology groupings, and 40 (87% [74–95]) of 46 for overall stage. Concordance between SYMPLIFY and WCISU was 87 (89% [95% CI 81–94]) of 98 for ICD-10, 62 (63% [53–73]) of 98 for ICD-O-3 morphology histological code, 78 (80% [70–87]) of 98 for ICD-O-3 broad morphology groupings, 37 (49% [38–61]) of 75 for TNM, and 81 (83% [74–90]) of 98 for overall stage (figure 3C; appendix pp 12–16).

**Figure 4:**
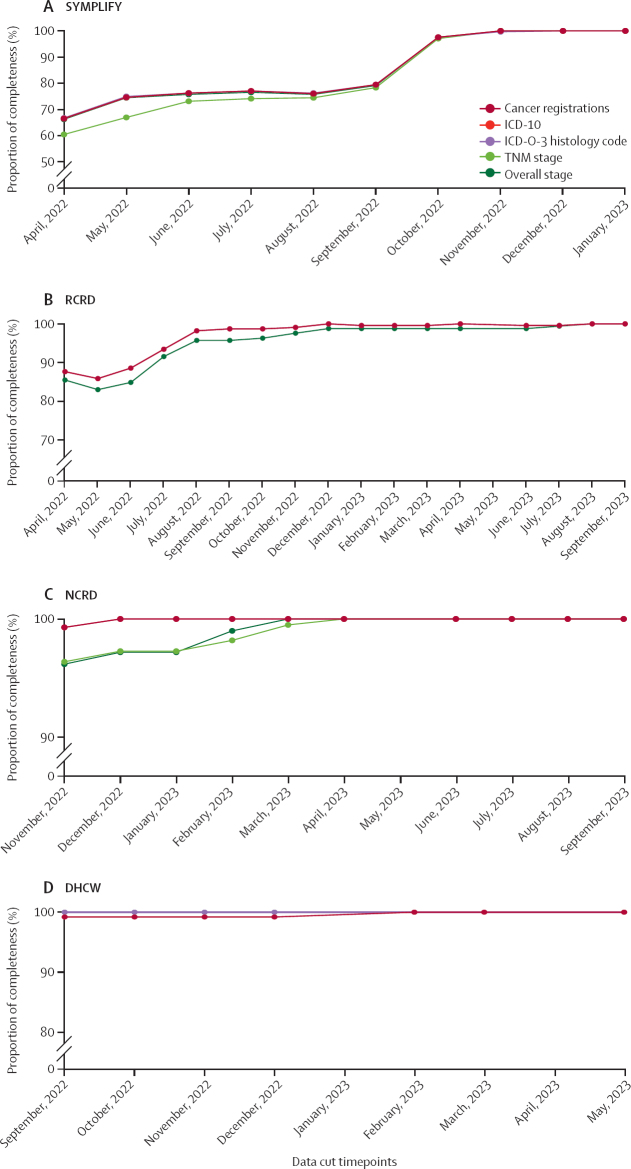
Timeliness of completeness of data fields for SYMPLIFY (A), RCRD (B), NCRD (C), and DHCW (D) data compared with the final dataset for each data source Number of cancer registrations, ICD-10, and ICD-O-3 histology code overlap in SYMPLIFY, RCRD, and NCRD. Number of cancer registrations and ICD-10 overlap with each other in DHCW, as do ICD-O-3 histology code and stage completion. DHCW=Digital Health and Care Wales. ICD-10=International Classification of Diseases, 10th Revision. ICD-O-3=International Classification of Diseases for Oncology, 3rd Edition. NCRD=National Cancer Registration Dataset. RCRD=Rapid Cancer Registration Dataset. WCISU=Welsh Cancer Intelligence and Surveillance Unit.

SYMPLIFY reached completeness of cancer registrations in November, 2022, approximately 12 months after study recruitment ended (figure 4A; appendix p 18). This dataset also reached full concordance in November, 2022, compared with the final most up-to-date dataset at 12 months post-enrolment for overall stage, and at 13 months post-enrolment for the remaining data fields (figure 4A; appendix p 4). NCRD and RCRD both reached completeness of cancer registrations compared with the corresponding final data cuts in December, 2022, at 13 months post-enrolment (figure 4B and C; appendix pp 4–5) Concordance was more than 90% for all data points at 12 months post-enrolment for NCRD. However, full concordance was reached at 13 months in December, 2022, for ICD-10, 16 months for stage in March, 2023, at 17 months for TNM stage in April, 2023, and at 21 months post-enrolment in August, 2023 for ICD-O-3 (appendix p 23). Similarly, the DHCW dataset reached full completeness of registrations at 15 months post-enrolment in February, 2023, while full concordance was reached at 10 months post-enrolment in September, 2023, for ICD-O-3 and overall stage, and 16 months in March, 2023 for ICD-10 (figure 4D; appendix p 5).

Among the cancers that were reported by NCRD but not SYMPLIFY, at the last NCRD data cut available in September, 2023, 30 (55%) of 55 were diagnosed within the 3-month post-enrolment period in SYMPLIFY. Among the 14 cancers reported by WCISU but not SYMPLIFY, six (43%) were diagnosed within the 3-month post-enrolment period (appendix p 24). There were 23 cancers reported in SYMPLIFY England that were not reported in NCRD at the final timepoint. Further investigations of these cancers showed that most (14 [61%] of 23) were included in the registry data cuts but had been excluded from our analysis for one of several reasons described in the appendix (p 25).

Analyses of discordant overall stage, ICD-10, and ICD-O-3 morphology groupings cases are available in the appendix (pp 6–7, 26–30).

## Discussion

Our study outlines promising findings in support of using cancer registry-based diagnostic data during cancer studies in England and Wales. Studies that could benefit from the use of registry-based diagnostic data include interventional studies and others that use the event of cancer diagnosis, diagnostic coding, and staging as endpoints. We highlight comparable validity and timeliness of outcome reporting to inform decisions to use locally collected or registry data. Commonly cited drawbacks of registry-based research are the timeliness of data entry into medical registries and the completeness of data fields.^5^ We show that data from central cancer registries and datasets of England and Wales exhibit similar timeliness and proportions of completed data fields to on-site prospectively collected data. This finding is promising given the substantial resource burden associated with data collection and the need for timely data in cancer research. We observed lower concordance between the on-site and registry data for stage, TNM, and precise morphology than for cancer site. However, improvements in concordance were observed for ICD-O-3 morphology and TNM staging when broader groupings were used, or individual stages considered. Thus, the level of detail necessary for each research question must be considered before selecting the method of outcome definition. Although the data evaluated are directly applicable to the UK setting, our study has findings that are potentially relevant to any health-care setting where cancer data are collected systematically and collated centrally, and might be used in place of study-specific collection at site.

To our knowledge, this is the first study to compare the validity and timeliness of cancer diagnosis data collected at study sites with centrally collected registry data in England and Wales over a wide range of cancers and since the inception of the National Cancer Registration and Analysis Service, in 2013. The broad inclusion criteria, which included all cancers, except non-melanoma skin cancers, recruitment from central and peripheral hospital sites, and interrogation of rapid, administrative, and gold-standard datasets afford a broad view of cancer data quality. A limitation to our study is that patients were recruited as the health system emerged from the COVID-19 pandemic, with cancer research teams at hospital sites depleted, which might have affected the completeness of data collection at site. Short length of follow-up is another limitation to the study, as we did not continue data collection at the site for the same amount of time as we conducted follow-up in the registry. Furthermore, we looked at data cuts on a monthly basis. Although this timeline is helpful for determining the speed at which data can become accessible for researchers, it is less useful for epidemiological studies, which investigate cancer cases over the course of many years.

Our study included patients who were diagnosed with cancer following referral to an urgent suspected cancer referral pathway, due to presenting with symptoms that could be linked to cancer. Cancer data for patients who are diagnosed via other means (eg, emergency presentation, or routine screening) might differ from what is presented here. Nonetheless, urgent suspected cancer referral pathways are the most common route through which UK patients are diagnosed, with nearly 40% of patients with cancer in the UK being diagnosed via these pathways.^19^ Thus, the data we present are representative of a large proportion of cancer diagnoses in England and Wales.

An important limitation of our study was in the investigation of cancer stage. Differences in staging exist depending on whether pathological or clinical staging is used. Pathological staging utilises tissues removed during surgery to determine the stage of the cancer, while clinical staging utilises information obtained before surgery, such as from imaging, blood samples, and biopsies.^20^ Research staff based at the SYMPLIFY sites collated the data available in the hospital record at the time the electronic case report form was completed. As such, it is possible that staging was based on imaging, pathological, clinical, or a combination of these methods. NCRD and WCISU reported an integrated stage using all information available, while the stage reported in RCRD is a combination of pre-treatment and pathological staging. Inconsistencies among the methods used to assign cancer stage might have affected the reported concordance. Notably, since we only compared concordance between the registry and on-site datasets and did not investigate which dataset was correct in cases of discordance, we can only report on how registry data quality compares in relation to the study dataset. Registry audits and internal checks would still be required to determine which data source is correct in cases of misalignment.

Multiple studies have reported varying cancer registry data quality internationally.^21^, ^22^, ^23^, ^24^, ^25^ A recent study investigated registrations of cancers diagnosed in England in 2012, and found that the rate of missing staging information ranged from 10·2% for lung cancers to 18·4% for prostate cancers, which is lower than what we observed in NCRD.^26^ Compared with the most recent United Kingdom and Ireland Association of Cancer Registries annual indicator report, we found higher staging completion for NCRD at 100% (95% CI 98–100) and WCISU at 100% (97–100), which was reported at 71·8% and 81·1%, respectively.^27^, ^28^ However, this higher staging completion could be due to the specific curation process that the data for this project underwent. Compared with these gold-standard cancer registries, completeness was lower at 43% (95% CI 34–53) for cancer stage in the DHCW dataset and 73% (66–78) for RCRD, compared with the English NCRD at 100% (98–100). Other studies that have investigated the data quality of cancer registries in England and Wales were done before 2013 and therefore have limited relevance to the current registry system.

We observed a substantial increase in completeness of data fields in WCISU and NCRD compared with DHCW and RCRD. Whereas completeness of data fields was 100% for ICD-O-3 morphology and stage in WCISU and NCRD, final stage completeness was lower in DHCW and RCRD at 43% and 73%, respectively. Missing data might have significant effects on study design and power, potentially resulting in the inability to answer the research question of interest. Rapid datasets, such as DHCW and RCRD, were developed for real-time surveillance activities requiring real-time data. Given their lower level of completeness compared with the gold-standard registries, their use in research might best be reserved for situations in which timeliness is more critical than completion and accuracy. Given the many checks that a gold-standard cancer registry undergoes before complete registration, it is also likely that the staging listed in cancer registries is more accurate than that collected earlier at site. In studies requiring overall stage and TNM staging, cancer registry data should be prioritised over site-collected data if time restrictions do not apply.

At 1 month following the completion of the SYMPLIFY dataset, NCRD reached maximal registration of cancers that were found in SYMPLIFY. This shows that national cancer registration processes can support efficient trial delivery and can be used as an alternative to locally collected data. However, registry support of efficient trial delivery is reliant on adequate resourcing of infrastructure and staff outside of the core national cancer registrations processes. The English registry was provided with resources and funding which allowed for accelerated data acquisition specifically for this project. Thus, we demonstrate that registry data can be obtained in a similar timeframe to on-site data, but this level of resourcing and speed might not always be available for studies. The differences observed in the data completeness and concordance between SYMPLIFY and registry datasets in part reflect that the English registry was resourced to support SYMPLIFY earlier and to a greater extent than was the Welsh registry. Furthermore, resource limitations led to WCISU only being able to investigate cancer cases that were confirmed in DHCW, which possibly affected case ascertainment numbers in this dataset. The process of obtaining data extracts from cancer registries might require many administrative steps, paperwork, and delays. Thus, national cancer registries should be engaged early and receive adequate resourcing to enable and equip them to provide timely data to support trial delivery. Even with additional funding and resources provided to registries for trial delivery, registry-based research remains an economic alternative to the costly and time-intensive process of collecting data on-site.

Although there were seemingly lower levels of concordance between the registry and on-site datasets for certain variables, namely overall stage, TNM stage, and ICD-O-3 morphology, these discordance rates must be considered in a broader context. Differences in staging could be due to differences in staging methodology, as previously mentioned, or due to differences in timing of data collection. For example, the SYMPLIFY case report form might have been completed before staging was complete, contributing to higher discordance rates for this variable. Furthermore, in our investigation of discordant cases, we highlight that many instances of discordant ICD-O-3 morphology groupings exist due to differences in the level of detail reported by the various datasets. Thus, determining the level of specificity necessary for a particular research question, and weighing the importance of factors such as speed of data acquisition and accuracy are important considerations for researchers thinking of engaging in registry-based research.

We found cancers registered in the national registries that were not reported in SYMPLIFY. Study sites were requested to review the records for participants at 3 months post-enrolment. Those that remained unresolved underwent another review at 9 months. Despite this, we found that 55% (n=30) of cancers reported in NCRD but not in SYMPLIFY, and 43% (n=6) cancers reported in WCISU but not in SYMPLIFY were diagnosed within the first 3 months following enrolment. This demonstrates a potential benefit of cancer registry data in research, which allows for more thorough follow-up. Further investigation is necessary to understand why these cancers were missed by SYMPLIFY sites.

We found that a small proportion of cancers in England that were reported in SYMPLIFY (9%, n=23) were not registered in NCRD at 22 months post-enrolment. After further investigation, we found that 14 (61%) of these cancers were in fact registered in the cancer registries, but either their date of diagnosis preceded enrolment of the patient in the study, or the ICD-10 code listed by NCRD made the cancer ineligible by our study criteria. There are strict international criteria regarding the date of diagnosis indicated in a cancer registry, to allow for comparability between registries, which might have contributed to the discrepancies observed. The remaining cancers were not identified in the registry records. Thus, a small subset of cancers might experience a longer lag time in the registration process. Previous studies have suggested that cancer registry data quality can vary based on cancer type, disease, demographic, and treatment factors.^22^, ^25^ Future research should investigate the effect of these factors on the data quality in these datasets.

We have demonstrated that the gold-standard national cancer registries of England and Wales have similar validity and timeliness to a prospective cohort study with moderate concordance. Our study contributes to ongoing research which supports the use of centralised registration processes to support study delivery in decentralised cancer research.

### Contributors

### Data sharing

De-identified individual-level patient data can be provided to researchers upon written request 24 months after publication of the Article. Please send enquiries to the corresponding author. A detailed proposal for how the data will be used is required to allow assessment of the application.

## Declaration of interests

BDN and MRM receive institutional research funding from GRAIL. BDN reports grants, honoraria, and consulting fees from the National Institute for Health and Care Research (NIHR), Cancer Research UK, Royal College of General Practitioners, and Multi-Cancer Early Detection Consortium. DH and DA report payment to their institution from National Health Service Wales Cancer Network for additional cancer registration officer time to expedite population-based cancer registration for this study. DH and DA report grants from Moondance Cancer Initiative. FDRN reports honoraria for occasional sessional payment for congress talks for AstraZeneca, Boehringer Ingelheim, Bristol Myers Squibb, and Pfizer. RP reports funding from GRAIL and National Health Service England for the completion of this study, as well as grants from Medical Research Council, NIHR, and the Medical Science Division at the University of Oxford and is a statistical editor for BMJ and BMJ Medicine. HK, KNK, SH, KR, YL, and HN were all employees of GRAIL at the time of the study. HK reports a leadership position with GRAIL. All other authors declare no competing interests.
