# Supplementary material for: Validity and timeliness of cancer diagnosis data collected during a prospective cohort study and reported by the English and Welsh cancer registries: a retrospective, comparative analysis
Source: Lancet Oncol. 2024 Nov;25(11):1476–86. doi: 10.1016/S1470-2045(24)00497-2 (PMC12979245; doi:10.1016/S1470-2045(24)00497-2)
Supplement: Supplementary appendix [file mmc1.pdf]

THE LANCET
Oncology

Supplementary appendix

This appendix formed part of the original submission and has been peer reviewed. We post it as supplied by the authors.

Supplement to: Jackson A, Virdee PS, Tonner S, et al. Validity and timeliness of cancer diagnosis data collected during a prospective cohort study and reported by the English and Welsh cancer registries: a retrospective, comparative analysis. *Lancet Oncol* 2024; published online Oct 9. https://doi.org/10.1016/S1470-2045(24)00497-2.

Appendix

Table of Contents

Figures ... 3

Figure S1. Number of cancers recorded in each dataset for the SYMPLIFY cohort over time ... 3

Figure S2. Number of cancers identified in both SYMPLIFY and the corresponding central dataset over time, indicating the population of cancers that was investigated for concordance between on-site and central datasets. ... 3

Figure S3. Timeliness of concordance of data fields compared to the final dataset for each data source (a) SYMPLIFY, (b) RCRD, (c) NCRD, and (d) DHCW datasets. ICD-10 and ICD-O-3 overlap in RCRD, while ICD-O-3 and stage overlap in DHCW. ... 4

Figure S4. Summary of staging concordance between SYMPLIFY and (a) RCRD, (b) NCRD, and (c) DHCW and WCISU, over the study period as a percentage of the total number of cancers considered for concordance in a given month. ... 6

Tables ... 8

Table S1. Proportion (%) of cancers (n) recorded in each dataset at each timepoint with complete data for (a) ICD-O-3, (b) TNM stage, and (c) stage. ... 8

Table S2. Cancers found in both SYMPLIFY and the corresponding dataset at each timepoint used in the concordance analysis. Proportions are derived from the denominator of 259 cancers recorded in the SYMPLIFY-England dataset for RCRD and NCRD datasets, and the 121 cancers recorded in the SYMPLIFY-Wales dataset for DHCW and WCISU. ... 11

Table S3. Concordance (%) between cancer data fields recorded for participants at SYMPLIFY study sites and also in the corresponding central datasets at each timepoint for (a) ICD-10, (b) ICD-O-3 4-digit morphology code, (c) ICD-O-3 broad morphology grouping, (d) TNM stage and (e) stage. ... 12

Table S4. Concordance (%) of T category, N category, M category, overall TNM stage, and stage for each cancer (n) recorded in both SYMPLIFY and the NCRD dataset at each timepoint Proportions are based on the total number of cancers present in both SYMPLIFY and NCRD that had TNM staging complete in both datasets. ... 17

Table S5. Timeliness of completion of each data field calculated as a proportion (%) of the total number of cancers in the final data cut for (a) SYMPLIFY, (b) RCRD, (c) NCRD, and (d) DHCW datasets. ... 18

Table S6. Timeliness of concordance of each data field calculated as a proportion (%) of the total number of cancers in the final data cut for (a) SYMPLIFY, (b) RCRD, (c) NCRD, and (d) DHCW datasets. ... 21

Table S7. Date of diagnosis for cancers found in (a) NCRD (n=55) and (b) WCISU (n=14) that were not found in SYMPLIFY at the last registry data cut available (September 2023 and July 2023, respectively). Displayed as the number and percentage of cancers that had a date of diagnosis in each time frame ... 24

Table S8. Investigation of cancers that were reported in SYMPLIFY-England but not reported in NCRD at the final time point in September 2023 (n=23). ... 25

Table S9. Summary of staging concordance and discordance between SYMPLIFY and (a) RCRD (b) NCRD, and (c) DHCW and WCISU, over the study period as the number and percentage of cancers that fall in each concordance grouping at each time point. 26

Table S10. Discordant ICD-10 cases based on corresponding cancer site groupings between SYMPLIFY and (a) RCRD, (b) NCRD, (c) DHCW, and (d) WCISU datasets at the final timepoint available for each dataset. 28

Table S11. Discordant ICD-O-3 cases based on corresponding morphology groupings between SYMPLIFY and (a) RCRD, (b) NCRD, and (c) DHCW, and (d) WCISU datasets at the final timepoint available for comparison for each dataset. 29

Table S12. ICD-O-3 broad morphology groupings...31

Figures

Figure S1. Number of cancers recorded in each dataset for the SYMPLIFY cohort over time.

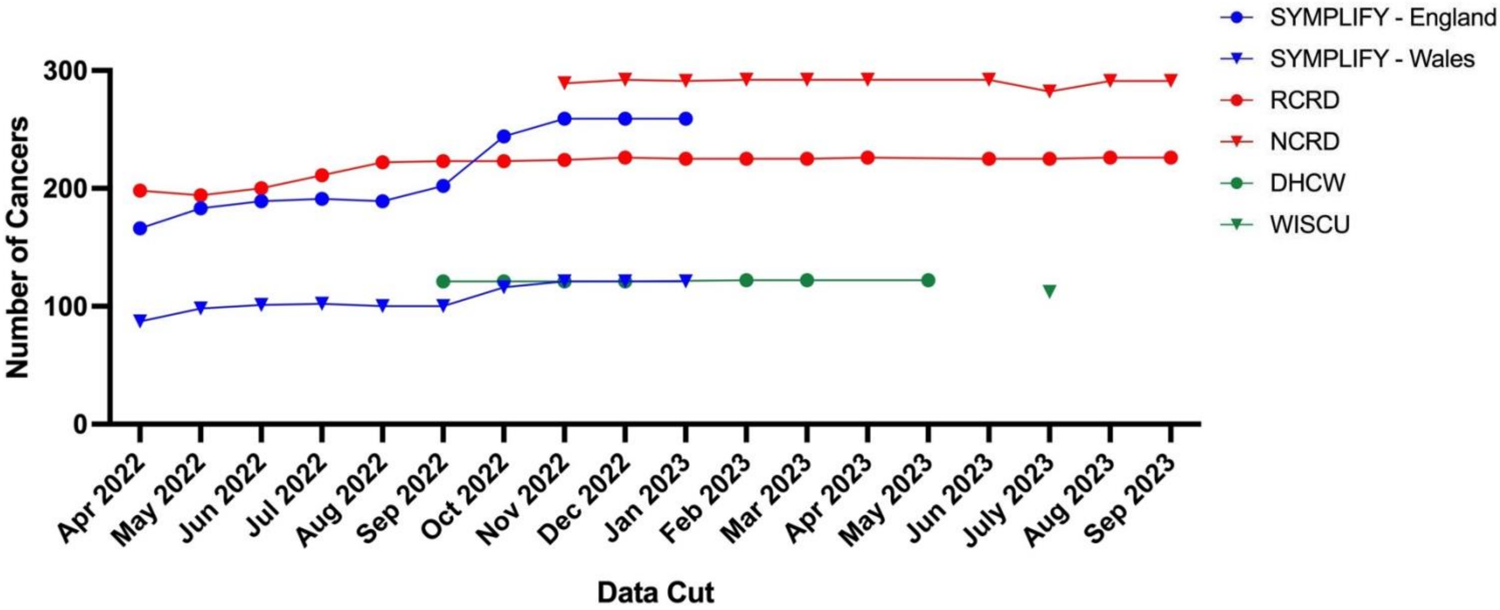

Figure S2. Number of cancers identified in both SYMPLIFY and the corresponding central dataset over time, indicating the population of cancers that was investigated for concordance between on-site and central datasets.

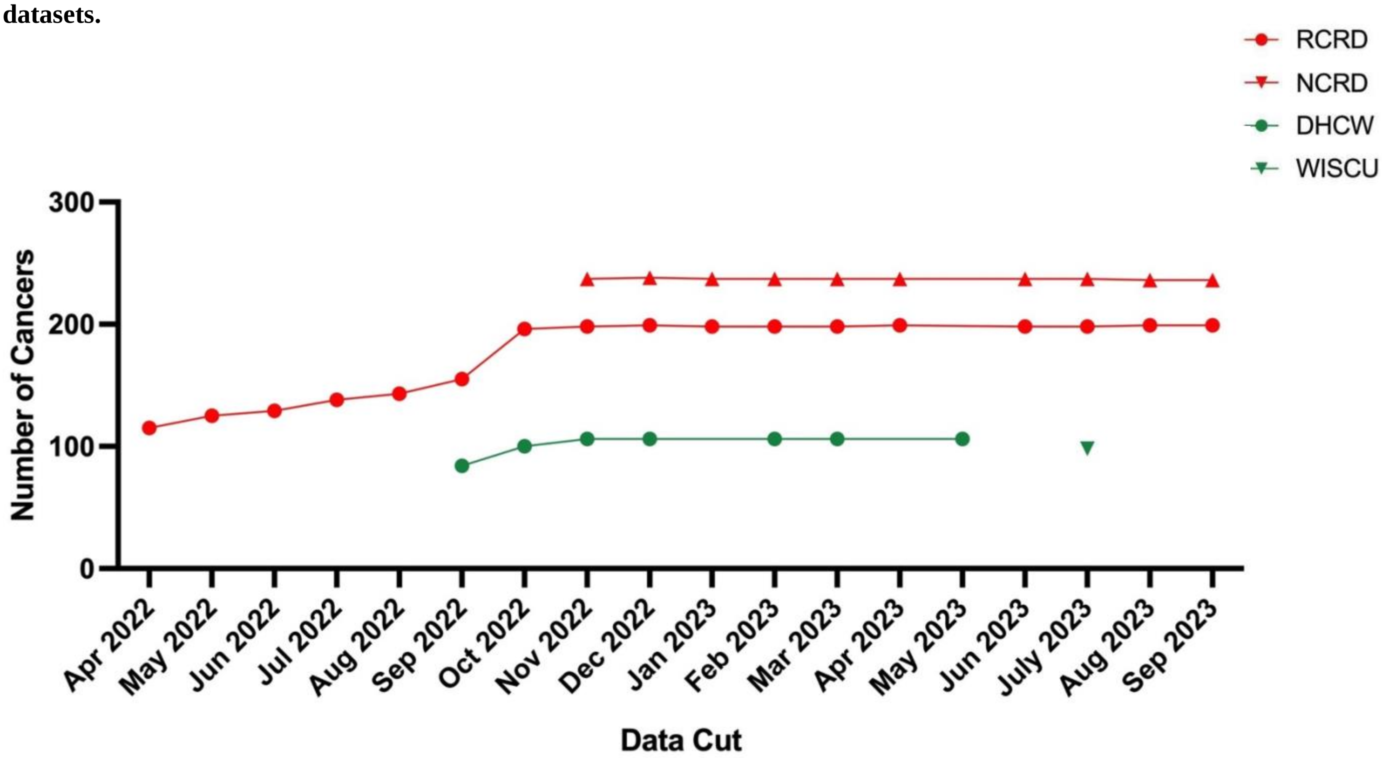

Figure S3. Timeliness of concordance of data fields compared to the final dataset for each data source (a) SYMPLIFY, (b) RCRD, (c) NCRD, and (d) DHCW datasets. ICD-10 and ICD-O-3 overlap in RCRD, while ICD-O-3 and stage overlap in DHCW.

a)

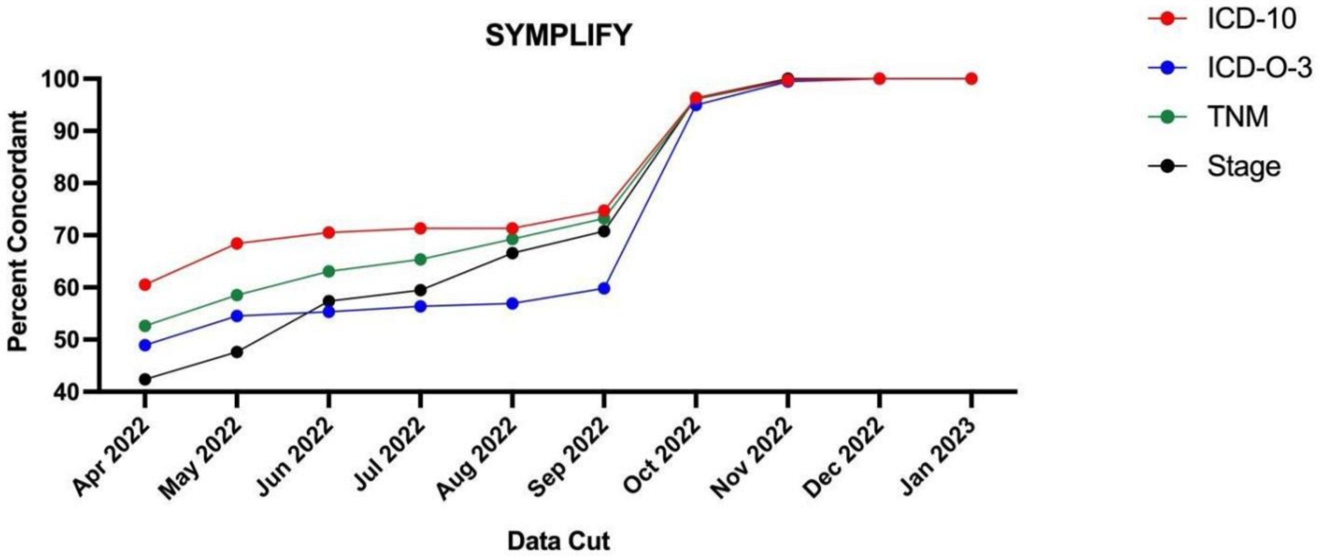

b)

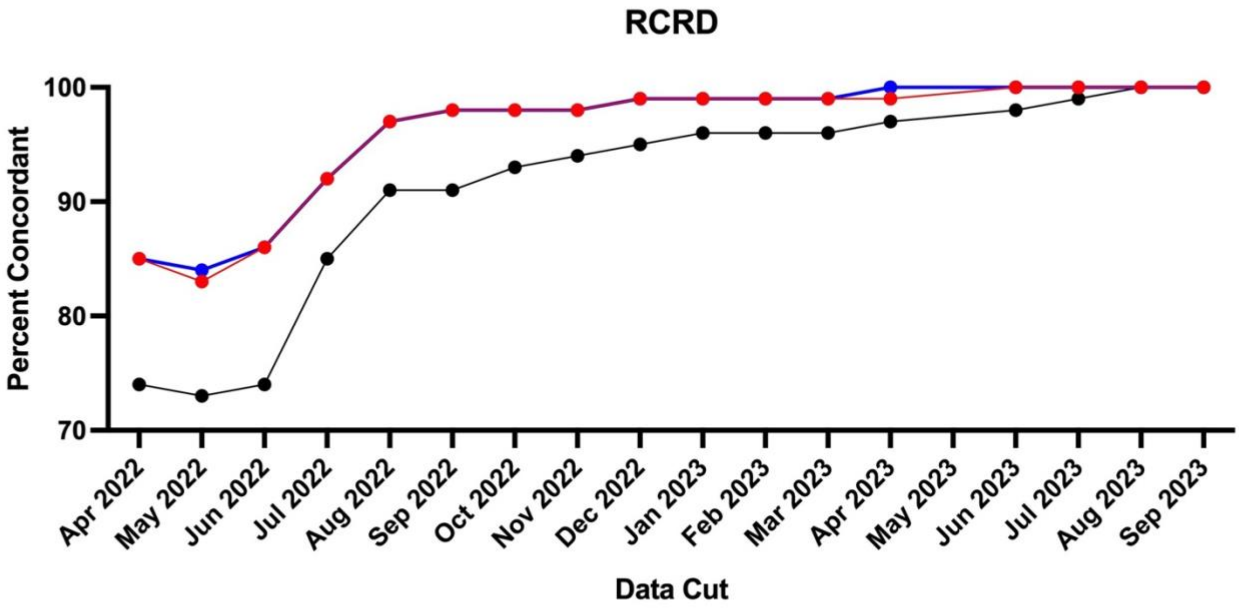

c)

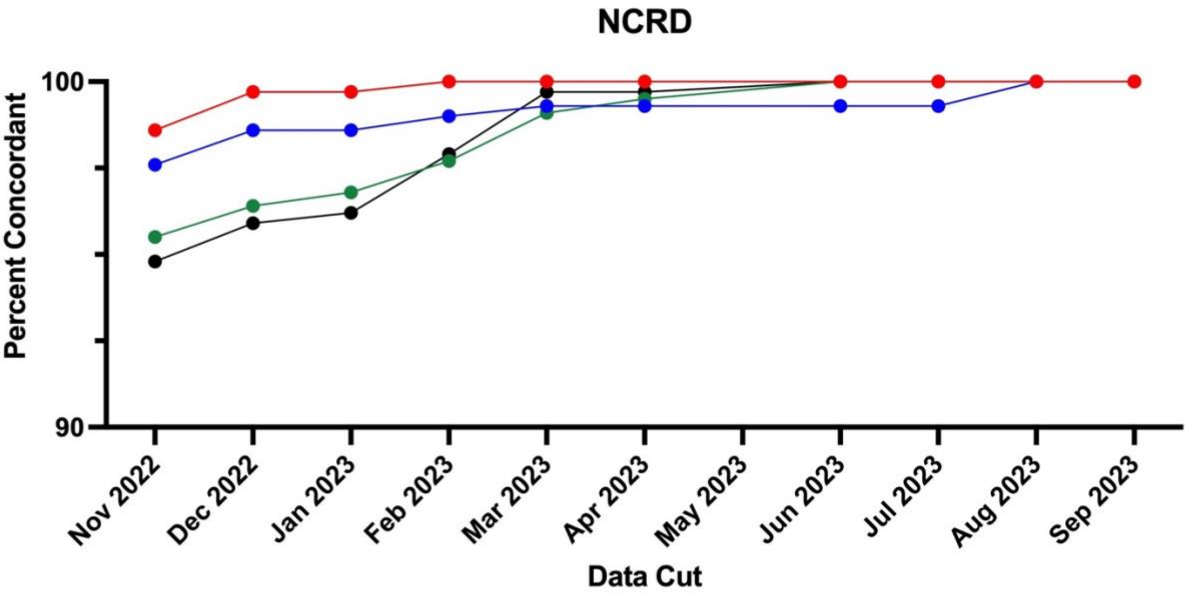
NCRD
100
90
Percent Concordant
Nov 2022
Dec 2022
Jan 2023
Feb 2023
Mar 2023
Apr 2023
May 2023
Jun 2023
Jul 2023
Aug 2023
Sep 2023
Data Cut

d)

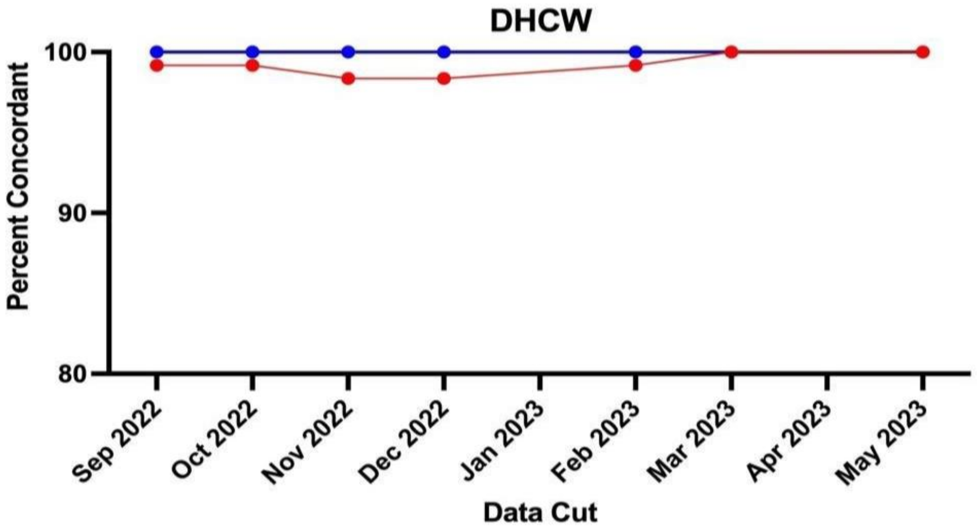
DHCW
100
90
80
Percent Concordant
Sep 2022
Oct 2022
Nov 2022
Dec 2022
Jan 2023
Feb 2023
Mar 2023
Apr 2023
May 2023
Data Cut

Figure S4. Summary of staging concordance between SYMPLIFY and (a) RCRD, (b) NCRD, and (c) DHCW and WCISU, over the study period as a percentage of the total number of cancers considered for concordance in a given month.

a)

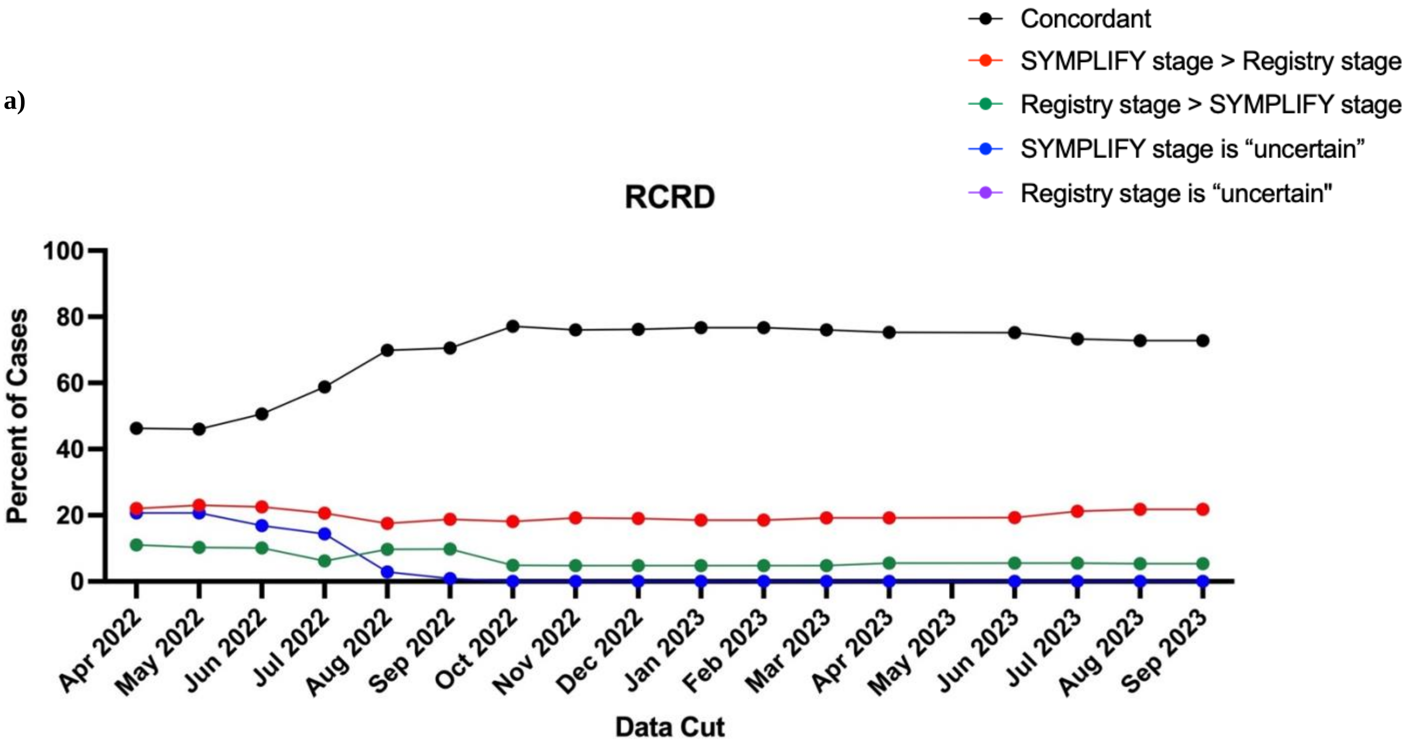

b)

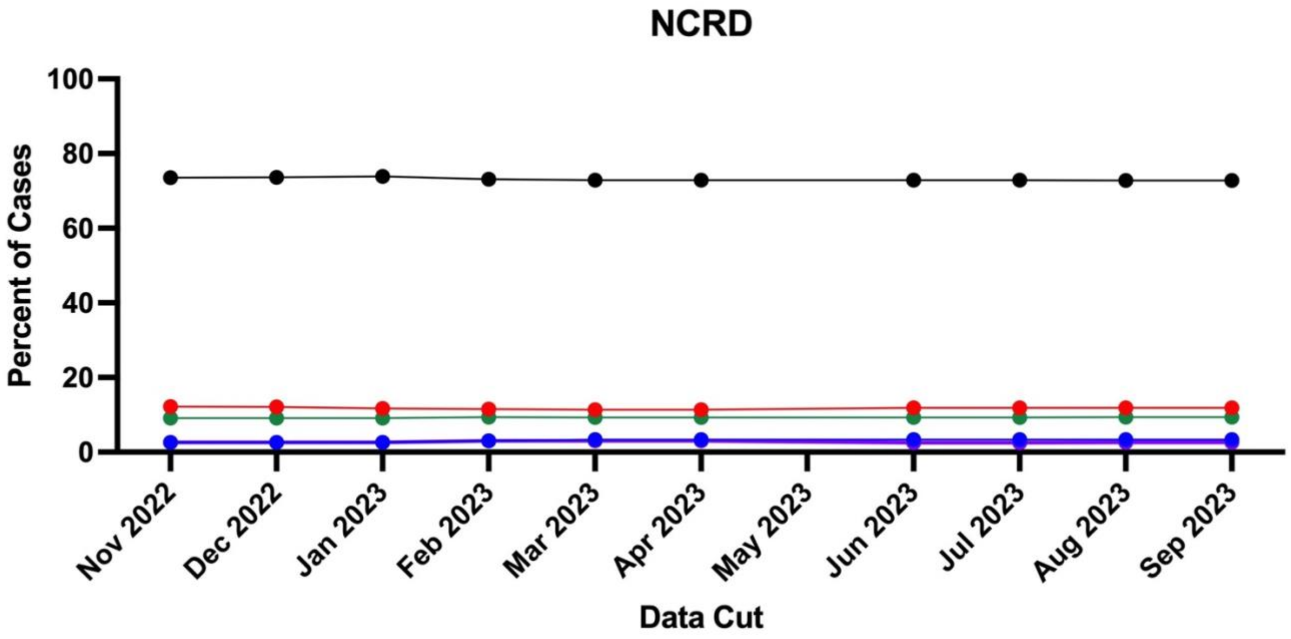

c)

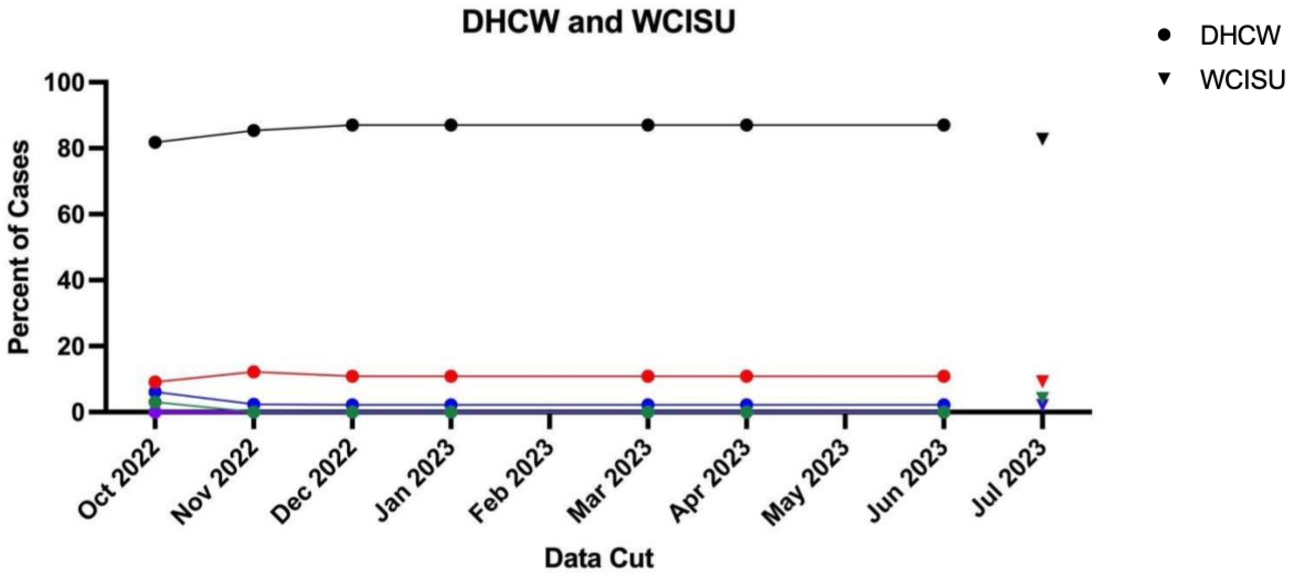
DHCW and WCISU
DHCW
WCISU
Percent of Cases
100
80
60
40
20
0
Oct 2022
Nov 2022
Dec 2022
Jan 2023
Feb 2023
Mar 2023
Apr 2023
May 2023
Jun 2023
Jul 2023
Data Cut

Tables

Table S1. Proportion (%) of cancers (n) recorded in each dataset at each timepoint with complete data for (a) ICD-O-3, (b) TNM stage, and (c) stage.

a) ICD-O-3

Data Cut	SYMPLIFY		RCRD % (95% CI, n)	NCRD % (95% CI, n)	DHCW % (95% CI, n)	WCISU % (95% CI, n)
	England % (95% CI, n)	Wales % (95% CI, n)				
**Apr 2022**	99 (96-100, 164)	100 (96-100, 87)	100 (98-100, 198)	-	-	-
**May 2022**	99 (96-100, 184)	100 (96-100, 98)	100 (98-100, 194)	-	-	-
**Jun 2022**	98 (95-100, 186)	100 (96-100, 101)	100 (98-100, 200)	-	-	-
**Jul 2022**	98 (95-100, 188)	100 (96-100, 102)	100 (98-100, 211)	-	-	-
**Aug 2022**	99 (96-100, 187)	100 (96-100, 100)	100 (98-100, 222)	-	-	-
**Sep 2022**	99 (96-100, 199)	100 (96-100, 100)	100 (98-100, 223)	-	84 (77-90, 102)	-
**Oct 2022**	98 (96-100, 251)	100 (97-100, 116)	100 (98-100, 223)	-	84 (77-90 102)	-
**Nov 2022**	98 (96-100, 255)	99 (95-100, 120)	100 (98-100, 224)	100 (99-100, 289)	84 (77-90, 102)	-
**Dec 2022**	98 (96-100, 255)	100 (97-100, 121)	100 (98-100, 226)	100 (99-100, 292)	84 (77-90, 102)	-
**Jan 2023**	98 (96-100, 255)	100 (97-100, 121)	100 (98-100, 225)	100 (99-100, 291)	-	-
**Feb 2023**	-	-	100 (98-100, 225)	100 (99-100, 292)	84 (76-90, 102)	-
**Mar 2023**	-	-	100 (98-100, 225)	100 (99-100, 292)	84 (76-90, 102)	-
**Apr 2023**	-	-	100 (98-100, 226)	100 (99-100, 292)	-	-
**May 2023**	-	-	-	-	84 (76-90, 102)	-
**Jun 2023**	-	-	100 (98-100, 225)	100 (99-100, 292)	-	-
**Jul 2023**	-	-	100 (98-100, 225)	100 (99-100, 292)	-	100 (97-100 112)
**Aug 2023**	-	-	100 (98-100, 226)	100 (99-100, 291)	-	-
**Sep 2023**	-	-	100 (98-100, 226)	100 (99-100, 291)	-	-

b) TNM stage

Data Cut	SYMPLIFY		RCRD % (95% CI, n)	NCRD % (95% CI, n)	DHCW % (95% CI, n)	WCISU % (95% CI, n)
	England % (95% CI, n)	Wales % (95% CI, n)				
**Apr 2022**	73 (65-79. 121)	71 (61-80, 62)	-	-	-	-
**May 2022**	72 (64-78, 133)	69 (59-78, 68)	-	-	-	-
**Jun 2022**	78 (71-83, 147)	72 (62-81, 73)	-	-	-	-
**Jul 2022**	79 (72-84, 150)	73 (63-81, 74)	-	-	-	-
**Aug 2022**	80 (73-85, 151)	74 (64-82, 74)	-	-	-	-
**Sep 2022**	81 (75-86, 163)	74 (64-82, 74)	-	-	-	-
**Oct 2022**	82 (77-87, 210)	72 (63-80, 84)	-	-	-	-
**Nov 2022**	83 (77-87, 214)	74 (65-81, 89)	-	74 (69-79, 214)	-	-
**Dec 2022**	83 (77-87, 214)	74 (65-81, 89)	-	74 (69-79, 216)	-	-
**Jan 2023**	83 (77-87, 214)	74 (65-81, 89)	-	74 (69-79, 216)	-	-
**Feb 2023**	-	-	-	75 (69-80, 218)	-	-
**Mar 2023**	-	-	-	76 (70-80, 221)	-	-
**Apr 2023**	-	-	-	76 (71-81, 222)	-	-
**May 2023**	-	-	-	-	-	-
**Jun 2023**	-	-	-	76 (71-81, 223)	-	-
**Jul 2023**	-	-	-	76 (71-81, 223)	-	77 (68-84, 86)
**Aug 2023**	-	-	-	76 (71-81, 222)	-	-
**Sep 2023**	-	-	-	76 (71-81, 222)	-	-

c) Stage

Data Cut	SYMPLIFY		RCRD % (95% CI, n)	NCRD % (95% CI, n)	DHCW % (95% CI, n)	WCISU % (95% CI, n)
	England % (95% CI, n)	Wales % (95% CI, n)				
**Apr 2022**	99 (97-100, 165)	100 (96-100, 87)	71 (64-77, 140)	-	-	-
**May 2022**	99 (97-100, 185)	100 (96-100, 98)	70 (63-76, 136)	-	-	-
**Jun 2022**	99 (96-100, 187)	100 (96-100, 101)	70 (63-76, 139)	-	-	-
**Jul 2022**	99 (96-100, 189)	100 (96-100, 102)	71 (64-77, 150)	-	-	-
**Aug 2022**	99 (97-100, 188)	100 (96-100, 100)	71 (64-77, 157)	-	-	-
**Sep 2022**	100 (97-100, 201)	100 (96-100, 100)	70 (64-76, 157)	-	44 (35-53, 53)	-
**Oct 2022**	100 (99-100, 255)	100 (97-100, 116)	71 (64-77, 158)	-	44 (35-53, 53)	-
**Nov 2022**	100 (99-100, 259)	100 (97-100, 121)	71 (65-77, 160)	97 (94-98, 279)	44 (35-53, 53)	-
**Dec 2022**	100 (99-100, 259)	100 (97-100, 121)	72 (65-77, 162)	97 (94-98, 282)	44 (35-53, 53)	-
**Jan 2023**	100 (99-100, 259)	100 (97-100, 121)	72 (66-78, 162)	97 (94-99, 282)	-	-
**Feb 2023**	-	-	72 (66-78, 162)	98 (96-99, 287)	43 (34-53, 53)	-
**Mar 2023**	-	-	72 (66-78, 162)	100 (98-100, 291)	43 (34-53, 53)	-
**Apr 2023**	-	-	72 (65-77, 162)	100 (98-100, 291)	-	-
**May 2023**	-	-	-	-	43 (34-53, 53)	-
**Jun 2023**	-	-	72 (66-78, 162)	100 (98-100, 291)	-	-
**Jul 2023**	-	-	72 (66-78, 163)	100 (98-100, 290)	-	100 (97-100, 112)
**Aug 2023**	-	-	73 (66-78, 164)	100 (98-100, 290)	-	-
**Sep 2023**	-	-	73 (66-78, 164)	100 (98-100, 290)	-	-

Table S2. Cancers found in both SYMPLIFY and the corresponding dataset at each timepoint used in the concordance analysis. Proportions are derived from the denominator of 259 cancers recorded in the SYMPLIFY-England dataset for RCRD and NCRD datasets, and the 121 cancers recorded in the SYMPLIFY-Wales dataset for DHCW and WCISU.

**Data Cut**	**Dataset**<br>n cancers in dataset (n patients with cancer, % total SYMPLIFY cancers)			
	**RCRD**	**NCRD**	**DHCW**	**WCISU**
**Apr 2022**	115 (115, 44)	-	-	-
**May 2022**	125 (125, 48)	-	-	-
**Jun 2022**	129 (129, 50)	-	-	-
**Jul 2022**	138 (138, 53)	-	-	-
**Aug 2022**	143 (143, 55)	-	-	-
**Sep 2022**	155 (155, 60)	-	84 (83, 69)	-
**Oct 2022**	196 (195, 76)	-	100 (99, 83)	-
**Nov 2022**	198 (197, 76)	237 (231, 92)	106 (105, 88)	-
**Dec 2022**	199 (198, 77)	238 (232, 92)	106 (105, 88)	-
**Jan 2023**	198 (197, 76)	237 (231, 92)	-	-
**Feb 2023**	198 (197, 76)	237 (231, 92)	106 (105, 88)	-
**Mar 2023**	198 (197, 76)	237 (231, 92)	106 (105, 88)	-
**Apr 2023**	199 (197, 77)	237 (231, 92)	-	-
**May 2023**	-	-	106 (105, 88)	-
**Jun 2023**	198 (196, 76)	237 (231, 92)	-	-
**Jul 2023**	198 (196, 76)	237 (231, 92)	-	98 (97, 81)
**Aug 2023**	199 (197, 77)	236 (230, 91)	-	-
**Sep 2023**	199 (197, 77)	236 (230, 91)	-	-

Table S3. Concordance (%) between cancer data fields recorded for participants at SYMPLIFY study sites and also in the corresponding central datasets at each timepoint for (a) ICD-10, (b) ICD-O-3 4-digit morphology code, (c) ICD-O-3 broad morphology grouping, (d) TNM stage and (e) stage.

a) **ICD-10**

Data Cut	RCRD<br>% (95% CI, n)	NCRD<br>% (95% CI, n)	DHCW<br>% (95% CI, n)	WCISU<br>% (95% CI, n)
**Apr 2022**	89 (81-94, 102)	-	-	-
**May 2022**	90 (84-95, 113)	-	-	-
**Jun 2022**	91 (85-96, 118)	-	-	-
**Jul 2022**	93 (87-96, 128)	-	-	-
**Aug 2022**	93 (88-97, 133)	-	-	-
**Sep 2022**	93 (88-96, 144)	-	95 (88-99, 80)	-
**Oct 2022**	95 (91-98, 187)	-	96 (90-99, 98)	-
**Nov 2022**	95 (92-98, 189)	96 (92-98, 227)	95 (89-98, 101)	-
**Dec 2022**	95 (92-98, 190)	96 (92-98, 228)	95 (89-98, 101)	-
**Jan 2023**	95 (92-98, 189)	96 (92-98, 227)	-	-
**Feb 2023**	95 (92-98, 189)	96 (92-98, 227)	95 (89-98, 101)	-
**Mar 2023**	95 (92-98, 189)	96 (92-98, 227)	96 (89-98, 102)	-
**Apr 2023**	95 (92-98, 190)	96 (92-98, 227)	-	-
**May 2023**	-	-	96 (91-99, 102)	-
**Jun 2023**	95 (92-98, 189)	96 (92-98, 227)	-	-
**Jul 2023**	95 (92-98, 189)	96 (92-98, 227)	-	89 (81-94, 87)
**Aug 2023**	95 (92-98, 190)	96 (92-98, 226)	-	-
**Sep 2023**	95 (92-98, 190)	96 (92-98, 226)	-	-

b) ICD-O-3 4-digit morphology code

Data Cut	RCRD<br>% (95% CI, n)	NCRD<br>% (95% CI, n)	DHCW<br>% (95% CI, n)	WCISU<br>% (95% CI, n)
**Apr 2022**	51 (42-61, 59)	-	-	-
**May 2022**	51 (42-60, 64)	-	-	-
**Jun 2022**	52 (43-61, 67)	-	-	-
**Jul 2022**	55 (46-64, 76)	-	-	-
**Aug 2022**	55 (46-63, 78)	-	-	-
**Sep 2022**	55 (47-63, 86)	-	47 (35-59, 35)	-
**Oct 2022**	67 (60-73, 130)	-	72 (62-81, 63)	-
**Nov 2022**	68 (61-74, 134)	60 (53-66, 139)	74 (63-82, 67)	-
**Dec 2022**	68 (61-74, 134)	60 (53-66, 140)	74 (64-83, 68)	-
**Jan 2023**	68 (60-74, 133)	60 (53-66, 139)	-	-
**Feb 2023**	68 (60-74, 133)	60 (53-66, 139)	74 (64-83, 68)	-
**Mar 2023**	68 (60-74, 133)	60 (53-66, 139)	74 (64-83, 68)	-
**Apr 2023**	68 (61-74, 134)	60 (53-66, 139)	-	-
**May 2023**	-	-	74 (64-83, 68)	-
**Jun 2023**	68 (60-74, 133)	60 (53-66, 139)	-	-
**Jul 2023**	68 (60-74, 133)	60 (53-66, 139)	-	63 (53-73, 62)
**Aug 2023**	67 (60-74, 133)	60 (53-66, 139)	-	-
**Sep 2023**	67 (60-74, 133)	60 (53-66, 139)	-	-

c) **ICD-O-3 broad morphology grouping**

Data Cut	RCRD<br>% (95% CI, n)	NCRD<br>% (95% CI, n)	DHCW<br>% (95% CI, n)	WCISU<br>% (95% CI, n)
**Apr 2022**	70 (60-78, 80)	-	-	-
**May 2022**	70 (62-78, 88)	-	-	-
**Jun 2022**	71 (62-78, 91)	-	-	-
**Jul 2022**	72 (63-79, 99)	-	-	-
**Aug 2022**	71 (63-79, 102)	-	-	-
**Sep 2022**	73 (65-78, 113)	-	55 (43-66, 41)	-
**Oct 2022**	83 (77-88, 162)	-	82 (72-89, 71)	-
**Nov 2022**	85 (79-89, 167)	83 (78-88, 194)	84 (74-90, 76)	-
**Dec 2022**	85 (79-90, 168)	83 (78-88, 195)	84 (75-91, 77)	-
**Jan 2023**	85 (79-89, 167)	83 (78-88, 194)	-	-
**Feb 2023**	85 (79-89, 167)	83 (78-88,194)	84 (75-91, 77)	-
**Mar 2023**	85 (79-89, 167)	83 (78-88, 194)	84 (75-91, 77)	-
**Apr 2023**	85 (79-90, 168)	83 (78-88, 194)	-	-
**May 2023**	-	-	84 (75-91, 77)	-
**Jun 2023**	85 (79-89, 167)	83 (78-88, 194)	-	-
**Jul 2023**	85 (79-89, 167)	83 (78-88, 194)	-	80 (70-87, 78)
**Aug 2023**	85 (79-90, 168)	83 (78-88, 193)	-	-
**Sep 2023**	85 (79-90, 168)	83 (78-88, 193)	-	-

d) TNM stage

Data Cut	RCRD<br>% (95% CI, n)	NCRD<br>% (95% CI, n)	DHCW<br>% (95% CI, n)	WCISU<br>% (95% CI, n)
**Apr 2022**	-	-	-	-
**May 2022**	-	-	-	-
**Jun 2022**	-	-	-	-
**Jul 2022**	-	-	-	-
**Aug 2022**	-	-	-	-
**Sep 2022**	-	-	-	-
**Oct 2022**	-	-	-	-
**Nov 2022**	-	50 (42-57, 86)	-	-
**Dec 2022**	-	50 (42-58, 87)	-	-
**Jan 2023**	-	50 (43-58, 87)	-	-
**Feb 2023**	-	51 (43-58, 88)	-	-
**Mar 2023**	-	51 (43-58, 89)	-	-
**Apr 2023**	-	51 (44-59, 90)	-	-
**May 2023**	-	-	-	-
**Jun 2023**	-	51 (43-58, 90)	-	-
**Jul 2023**	-	51 (43-58, 90)	-	49 (38-61, 37)
**Aug 2023**	-	51 (44-59, 90)	-	-
**Sep 2023**	-	51 (44-59, 90)	-	-

e) Stage

Data Cut	RCRD<br>% (95% CI, n)	NCRD<br>% (95% CI, n)	DHCW<br>% (95% CI, n)	WCISU<br>% (95% CI, n)
**Apr 2022**	46 (35-58, 38)	-	-	-
**May 2022**	46 (35-57, 40)	-	-	-
**Jun 2022**	51 (40-61, 45)	-	-	-
**Jul 2022**	59 (48-69, 57)	-	-	-
**Aug 2022**	70 (60-79, 72)	-	-	-
**Sep 2022**	71 (61-79, 79)	-	82 (65-93, 27)	-
**Oct 2022**	77 (69-84, 111)	-	85 (71-94, 35)	-
**Nov 2022**	76 (68-83, 111)	73 (67-79, 169)	87 (74-95, 40)	-
**Dec 2022**	76 (68-83, 112)	74 (67-79, 170)	87 (74-95, 40)	-
**Jan 2023**	77 (69-83, 112)	74 (68-79, 170)	-	-
**Feb 2023**	77 (69-83, 112)	73 (67-79, 171)	87 (74-95, 40)	-
**Mar 2023**	76 (68-83, 111)	73 (67-78, 172)	87 (74-95, 40)	-
**Apr 2023**	75 (68-82, 110)	73 (67-78, 172)	-	-
**May 2023**	-	-	87 (74-95, 40)	-
**Jun 2023**	75 (67-82, 109)	73 (67-78, 172)	-	-
**Jul 2023**	73 (65-80, 107)	73 (67-78, 172)	-	83 (74-90, 81)
**Aug 2023**	73 (65-80, 107)	73 (67-78, 171)	-	-
**Sep 2023**	73 (65-80, 107)	73 (67-78, 171)	-	-

Table S4. Concordance (%) of T category, N category, M category, overall TNM stage, and stage for each cancer (n) recorded in both SYMPLIFY and the NCRD dataset at each timepoint Proportions are based on the total number of cancers present in both SYMPLIFY and NCRD that had TNM staging complete in both datasets.

Data Cut	T category<br>% (95% CI, n)	N category<br>% (95% CI, n)	M category<br>% (95% CI, n)	TNM stage<br>% (95% CI, n)	Stage<br>% (95% CI, n)
**Nov 2022**	73 (66-80, 127)	77 (70-83, 134)	91 (86-95, 158)	50 (42-57, 86)	73 (67-79, 169)
**Dec 2022**	74 (66-80, 128)	78 (71-84, 135)	91 (86-95, 159)	50 (42-58, 87)	74 (67-79, 170)
**Jan 2023**	74 (67-80, 128)	78 (71-84, 135)	91 (86-95, 158)	50 (43-58, 87)	74 (68-79, 170)
**Feb 2023**	74 (67-80, 129)	78 (71-84, 136)	91 (86-95, 159)	51 (43-58, 88)	73 (67-79, 171)
**Mar 2023**	74 (67-81, 130)	78 (71-84, 137)	91 (86-95, 160)	51 (43-58, 89)	73 (67-78, 172)
**Apr 2023**	74 (67-81, 131)	78 (72-84, 138)	91 (86-95, 161)	51 (44-59, 90)	73 (67-78, 172)
**May 2023**	-	-	-	-	-
**Jun 2023**	74 (67-80, 131)	78 (71-84, 138)	91 (86-95, 161)	51 (43-58, 90)	73 (67-78, 172)
**Jul 2023**	74 (67-80, 131)	78 (71-84, 138)	91 (86-95, 161)	51 (43-58, 90)	73 (67-78, 172)
**Aug 2023**	74 (67-80, 130)	78 (71-84, 138)	91 (86-95, 160)	51 (44-59, 90)	73 (67-78, 171)
**Sep 2023**	74 (67-80, 130)	78 (71-84, 138)	91 (86-95, 160)	51 (44-59, 90)	73 (67-78, 171)

Table S5. Timeliness of completion of each data field calculated as a proportion (%) of the total number of cancers in the final data cut for (a) SYMPLIFY, (b) RCRD, (c) NCRD, and (d) DHCW datasets.

a) SYMPLIFY

Data Cut	Data Field<br>% (95% CI, n)				
	**Cancers**	**ICD-10**	**ICD-O-3**	**TNM stage**	**Stage**
**Apr 2022**	67 (62-71, 253)	67 (62-71, 253)	67 (62-72, 251)	60 (55-66, 185)	66 (61-71, 252)
**May 2022**	75 (70-79, 284)	75 (70-79, 284)	75 (70-79, 282)	67 (61-72, 205)	74 (70-79, 283)
**Jun 2022**	76 (72-81, 290)	76 (72-81, 290)	76 (72-81, 287)	73 (68-78, 224)	76 (71-80, 288)
**Jul 2022**	77 (73-81, 293)	77 (73-81, 293)	77 (73-81, 290)	74 (69-79, 227)	77 (72-81, 291)
**Aug 2022**	76 (71-80, 289)	76 (71-80, 289)	76 (72-81, 287)	75 (69-79, 228)	76 (71-80, 288)
**Sep 2022**	79 (75-83, 302)	79 (75-83, 302)	80 (75-83, 299)	78 (73-83, 240)	79 (75-83, 301)
**Oct 2022**	98 (96-99, 371)	98 (96-99, 371)	98 (96-99, 367)	97 (94-99, 297)	98 (96-99, 371)
**Nov 2022**	100 (99-100, 380)	100 (99-100, 380)	100 (99-100, 375)	100 (99-100, 306)	100 (99-100, 380)
**Dec 2022**	100 (99-100, 380)	100 (99-100, 380)	100 (99-100, 376)	100 (99-100, 306)	100 (99-100, 380)
**Jan 2023**	100 (99-100, 380)	100 (99-100, 380)	100 (99-100, 376)	100 (99-100, 306)	100 (99-100, 380)

b) RCRD

Data Cut	Data Field % (95% CI, n)				
	**Cancers**	**ICD-10**	**ICD-O-3**	**TNM stage**	**Stage**
**Apr 2022**	88 (83-92, 198)	88 (83-92, 198)	88 (83-92, 198)	-	85 (79-90, 140)
**May 2022**	86 (81-90, 194)	86 (81-90, 194)	86 (81-90, 194)	-	83 (76-88, 136)
**Jun 2022**	88 (84-92, 200)	88 (84-92, 200)	88 (84-92, 200)	-	85 (78-90, 139)
**Jul 2022**	93 (89-96, 211)	93 (89-96, 211)	93 (89-96, 211)	-	91 (86-95, 150)
**Aug 2022**	98 (96-100, 222)	98 (96-100, 222)	98 (96-100, 222)	-	96 (91-98, 157)
**Sep 2022**	99 (96-100, 223)	99 (96-100, 223)	99 (96-100, 223)	-	96 (91-98, 157)
**Oct 2022**	99 (96-100, 223)	99 (96-100, 223)	99 (96-100, 223)	-	96 (92-99, 158)
**Nov 2022**	99 (97-100, 224)	99 (97-100, 224)	99 (97-100, 224)	-	98 (94-99, 160)
**Dec 2022**	100 (98-100, 226)	100 (98-100, 226)	100 (98-100, 226)	-	99 (96-100, 162)
**Jan 2023**	100 (98-100, 225)	100 (98-100, 225)	100 (98-100, 225)	-	99 (96-100, 162)
**Feb 2023**	100 (98-100, 225)	100 (98-100, 225)	100 (98-100, 225)	-	99 (96-100, 162)
**Mar 2023**	100 (98-100, 225)	100 (98-100, 225)	100 (98-100, 225)	-	99 (96-100, 162)
**Apr 2023**	100 (98-100, 226)	100 (98-100, 226)	100 (98-100, 226)	-	99 (96-100, 162)
**May 2023**	-	-	-	-	-
**Jun 2023**	100 (98-100, 225)	100 (98-100, 225)	100 (98-100, 225)	-	99 (96-100, 162)
**Jul 2023**	100 (98-100, 225)	100 (98-100, 225)	100 (98-100, 225)	-	99 (97-100, 163)
**Aug 2023**	100 (98-100, 226)	100 (98-100, 226)	100 (98-100, 226)	-	100 (98-100, 164)
**Sep 2023**	100 (98-100, 226)	100 (98-100, 226)	100 (98-100, 226)	-	100 (98-100, 164)

c) NCRD

Data Cut	Data Field<br>% (95% CI, n)				
	Cancers	ICD-10	ICD-O-3	TNM stage	Stage
**Nov 2022**	99 (98-100, 289)	99 (98-100, 289)	99 (98-100, 289)	96 (93-98, 214)	96 (93-98, 279)
**Dec 2022**	100 (99-100, 292)	100 (99-100, 292)	100 (99-100, 292)	97 (94-99, 216)	97 (95-99, 282)
**Jan 2023**	100 (99-100, 291)	100 (99-100, 291)	100 (99-100, 291)	97 (94-99, 216)	97 (95-99, 282)
**Feb 2023**	100 (99-100, 292)	100 (99-100, 292)	100 (99-100, 292)	98 (95-100, 218)	99 (97-100, 287)
**Mar 2023**	100 (99-100, 292)	100 (99-100, 292)	100 (99-100, 292)	100 (98-100, 221)	100 (99-100, 291)
**Apr 2023**	100 (99-100, 292)	100 (99-100, 292)	100 (99-100, 292)	100 (98-100, 222)	100 (99-100, 291)
**May 2023**	-	-	-	-	-
**Jun 2023**	100 (99-100, 292)	100 (99-100, 292)	100 (99-100, 292)	100 (98-100, 223)	100 (99-100, 291)
**Jul 2023**	100 (99-100, 292)	100 (99-100, 292)	100 (99-100, 292)	100 (98-100, 223)	100 (99-100, 291)
**Aug 2023**	100 (99-100, 291)	100 (99-100, 291)	100 (99-100, 291)	100 (98-100, 222)	100 (99-100, 290)
**Sep 2023**	100 (99-100, 291)	100 (99-100, 291)	100 (99-100, 291)	100 (98-100, 222)	100 (99-100, 290)

d) DHCW

Data Cut	Data Field<br>% (95% CI, n)				
	Cancers	ICD-10	ICD-O-3	TNM stage	Stage
**Sep 2022**	99 (96-100, 121)	99 (96-100, 121)	100 (96-100, 102)	-	100 (93-100, 53)
**Oct 2022**	99 (96-100, 121)	99 (96-100, 121)	100 (96-100, 102)	-	100 (93-100, 53
**Nov 2022**	99 (96-100, 121)	99 (96-100, 121)	100 (96-100, 102)	-	100 (93-100, 53
**Dec 2022**	99 (96-100, 121)	99 (96-100, 121)	100 (96-100, 102)	-	100 (93-100, 53
**Jan 2023**	-	-	-	-	-
**Feb 2023**	100 (97-100, 122)	100 (97-100, 122)	100 (96-100, 102)	-	100 (93-100, 53
**Mar 2023**	100 (97-100, 122)	100 (97-100, 122)	100 (96-100, 102)	-	
**Apr 2023**	-	-	-	-	-
**May 2023**	100 (97-100, 122)	100 (97-100, 122)	100 (96-100, 102)	-	100 (93-100, 53

Table S6. Timeliness of concordance of each data field calculated as a proportion (%) of the total number of cancers in the final data cut for (a) SYMPLIFY, (b) RCRD, (c) NCRD, and (d) DHCW datasets.

a) **SYMPLIFY**

**Data Cut**	**Data Field** % (95% CI, n)			
	**ICD-10**	**ICD-O-3**	**TNM stage**	**Stage**
**Apr 2022**	61 (55-65, 230)	49 (44-54, 184)	53 (47-58, 161)	42 (37-48, 161)
**May 2022**	68 (63-73, 260)	55 (49-60, 205)	58 (53-64, 179)	48 (43-53, 181)
**Jun 2022**	71 (66-75, 268)	55 (50-60, 208)	63 (57-68, 193)	57 (52-62, 218)
**Jul 2022**	71 (66-76, 271)	56 (51-61, 212)	65 (60-71, 200)	59 (54-64, 226)
**Aug 2022**	71 (66-76, 271)	57 (52-62, 214)	69 (64-74, 212)	67 (62-71, 253)
**Sep 2022**	75 (70-79, 284)	60 (55-65, 225)	73 (68-78, 224)	71 (66-75, 269)
**Oct 2022**	96 (94-98, 366)	95 (92-97, 357)	96 (93-98, 294)	96 (94-98, 366)
**Nov 2022**	100 (99-100, 379)	99 (98-100, 374)	100 (98-100, 305)	100 (99-100, 380)
**Dec 2022**	100 (99-100, 380)	100 (99-100, 376)	100 (99-100, 306)	100 (99-100, 380)
**Jan 2023**	100 (99-100, 380)	100 (99-100, 376)	100 (99-100, 306)	100 (99-100, 380)

b) **RCRD**

**Data Cut**	**Data Field** % (95% CI, n)			
	**ICD-10**	**ICD-O-3**	**TNM stage**	**Stage**
**Apr 2022**	85 (79-89, 191)	85 (80-89, 192)	-	74 (66-80, 121)
**May 2022**	83 (78-88, 188)	84 (78-88, 189)	-	73 (65-79, 119)
**Jun 2022**	86 (81-90, 195)	86 (81-90, 195)	-	74 (67-81, 122)
**Jul 2022**	92 (87-95, 207)	92 (87-95, 207)	-	85 (79-90, 140)
**Aug 2022**	97 (94-99, 219)	97 (94-99, 219)	-	91 (85-95, 149)
**Sep 2022**	98 (96-100, 222)	98 (96-100, 222)	-	91 (86-95, 150)
**Oct 2022**	98 (96-100, 222)	98 (96-100, 222)	-	93 (88-96, 152)
**Nov 2022**	98 (96-100, 222)	98 (96-100, 222)	-	94 (89-97, 154)
**Dec 2022**	99 (97-100, 224)	99 (97-100, 224)	-	95 (91-98, 156)
**Jan 2023**	99 (97-100, 224)	99 (97-100, 224)	-	96 (91-98, 157)
**Feb 2023**	99 (97-100, 224)	99 (97-100, 224)	-	96 (91-98, 157)
**Mar 2023**	99. (97-100, 224)	99 (97-100, 224)	-	96 (92-99, 158)
**Apr 2023**	99 (98-100, 224)	100 (98-100, 225)	-	97 (93-99, 159)
**May 2023**	-	-	-	-
**Jun 2023**	100 (98-100, 225)	100 (98-100, 225)	-	98 (94-99, 160)
**Jul 2023**	100 (98-100, 225)	100 (98-100, 225)	-	99 (97-100, 163)
**Aug 2023**	100 (98-100, 226)	100 (98-100, 226)	-	100 (98-100, 164)
**Sep 2023**	100 (98-100, 226)	100 (98-100, 226)	-	100 (98-100, 164)

c) **NCRD**

**Data Cut**	**Data Field** % (95% CI, n)			
	**ICD-10**	**ICD-O-3**	**TNM stage**	**Stage**
**Nov 2022**	99 (97-100, 287)	98 (95-99, 284)	95 (92-98, 212)	95 (92-97, 275)
**Dec 2022**	100 (98-100, 290)	99 (97-100, 287)	96 (93-98, 214)	96 (93-98, 278)
**Jan 2023**	100 (98-100, 290)	99 (97-100, 287)	97 (94-99, 215)	96 (93-98, 279)
**Feb 2023**	100 (99-100, 291)	99 (97-100, 288)	98 (95-99, 217)	98 (96-99, 284)
**Mar 2023**	100 (99-100, 291)	99 (98-100, 289)	99 (97-100, 220)	100 (98-100, 289)
**Apr 2023**	100 (99-100, 291)	99 (98-100, 289)	100 (98-100, 221)	100 (98-100, 289)
**May 2023**	-	-	-	-
**Jun 2023**	100 (99-100, 291)	99 (98-100, 289)	100 (98-100, 222)	100 (99-100, 290)
**Jul 2023**	100 (99-100, 291)	99 (98-100, 289)	100 (98-100, 222)	100 (99-100, 290)
**Aug 2023**	100 (99-100, 291)	100 (99-100, 291)	100 (98-100, 222)	100 (99-100, 290)
**Sep 2023**	100 (99-100, 291)	100 (99-100, 291)	100 (98-100, 222)	100 (99-100, 290)

d) **DHCW**

**Data Cut**	**Data Field** % (95% CI, n)		
	**ICD-10**	**ICD-O-3**	**Stage**
**Sep 2022**	99 (96-100, 121)	100 (96-100, 102)	100 (93-100, 53)
**Oct 2022**	99 (96-100, 121)	100 (96-100, 102)	100 (93-100, 53)
**Nov 2022**	98 (94-100, 120)	100 (96-100, 102)	100 (93-100, 53)
**Dec 2022**	98 (94-100, 120)	100 (96-100, 102)	100 (93-100, 53)
**Jan 2023**	-	-	-
**Feb 2023**	99 (96-100, 121)	100 (96-100, 102)	100 (93-100, 53)
**Mar 2023**	100 (97-100, 122)	100 (96-100, 102)	100 (93-100, 53)
**Apr 2023**	-	-	-
**May 2023**	100 (97-100, 122)	100 (96-100, 102)	100 (93-100, 53)

Table S7. Date of diagnosis for cancers found in (a) NCRD (n=55) and (b) WCISU (n=14) that were not found in SYMPLIFY at the last registry data cut available (September 2023 and July 2023, respectively). Displayed as the number and percentage of cancers that had a date of diagnosis in each time frame.

a) NCRD

**Time Frame**	**Number of cancers diagnosed % (n)**	**Cumulative diagnoses % (n)**
**< 3 months post-enrolment**	55 (30)	55 (30)
**3-6 months post-enrolment**	31 (17)	85 (47)
**6-9 months post-enrolment**	15 (8)	100 (55)

b) WCISU

**Time Frame**	**Number of cancers diagnosed % (n)**	**Cumulative diagnoses % (n)**
**< 3 months post-enrolment**	43 (6)	43 (6)
**3-6 months post-enrolment**	36 (5)	79 (11)
**6-9 months post-enrolment**	21 (3)	100 (14)

Table S8. Investigation of cancers that were reported in SYMPLIFY-England but not reported in NCRD at the final time point in September 2023 (n=23).

Outcome of Investigation	Number of cancers % (n)	Additional Notes
**Cancer recorded in NCRD**	61 (14)	13 cancers were diagnosed pre-enrolment according to the date of diagnosis in NCRD<br><br>1 cancer in NCRD had an ineligible ICD-10 code resulting in its exclusion from the analysis
**Cancer not recorded in NCRD**	39 (9)	6 cases involved NCRD reporting historical cancers, but no current cancer diagnoses related to those reported in SYMPLIFY-England<br><br>2 cases involved a patient with 3 cancers reported in SYMPLIFY-England but only 1 reported in NCRD<br><br>1 case involved no cancer diagnoses being reported in NCRD

Table S9. Summary of staging concordance and discordance between SYMPLIFY and (a) RCRD (b) NCRD, and (c) DHCW and WCISU, over the study period as the number and percentage of cancers that fall in each concordance grouping at each time point.

a) RCRD

Data Cut	Total Cancers Considered	Concordant % (95% CI, n)	Discordant: SYMPLIFY stage > registry stage % (95% CI, n)	Discordant: Registry stage > SYMPLIFY stage % (95% CI, n)	Discordant: SYMPLIFY stage is "uncertain" % (95% CI, n)	Discordant: Registry stage is "uncertain" % (95% CI, n)
**Apr 2022**	82	46 (35-58, 38)	22 (14-32, 18)	11 (5-20, 9)	21 (13-31, 17)	0 (0-4, 0)
**May 2022**	87	46 (35-57, 40)	23 (15-33, 20)	10 (5-19, 9)	21 (13-31, 18)	0 (0-4, 0)
**Jun 2022**	89	51 (40-61, 45)	22 (14-33, 20)	10 (5-18, 9)	17 (10-26, 15)	0 (0-4, 0)
**Jul 2022**	97	59 (48-69, 57)	21 (13-30, 20)	6 (2-13, 6)	14 (8-23, 14)	0 (0-4, 0)
**Aug 2022**	103	70 (60-79, 72)	17 (11-26, 18)	10 (5-17, 10)	3 (1-8, 3)	0 (0-4, 0)
**Sep 2022**	112	71 (61-79, 79)	19 (12-27, 21)	10 (5-17, 11)	1 (0-5, 1)	0 (0-3, 0)
**Oct 2022**	144	77 (69-84, 111)	18 (12-25, 26)	4.9 (2-10, 7)	0 (0-3, 0)	0 (0-3, 0)
**Nov 2022**	146	76 (68-83, 111)	19 (13-27, 28)	5 (2-10, 7)	0 (0-2, 0)	0 (0-2, 0)
**Dec 2022**	147	76 (68-83, 112)	19 (13-26, 28)	5 (2-10, 7)	0 (0-2, 0)	0 (0-2, 0)
**Jan 2023**	146	77 (69-83, 112)	18 (13-26, 27)	5 (2-10, 7)	0 (0-2, 0)	0 (0-2, 0)
**Feb 2023**	146	77 (69-83, 112)	18 (13-26, 27)	5 (2-10, 7)	0 (0-2, 0)	0 (0-2, 0)
**Mar 2023**	146	76 (68-83, 111)	19 (13-27, 28)	5 (0-10, 7)	0 (0-2, 0)	0 (0-2, 0)
**Apr 2023**	146	75 (68-82, 110)	19 (13-27, 28)	5 (2-11, 8)	0 (0-2, 0)	0 (0-2, 0)
**May 2023**	-	-	-	-	-	-
**Jun 2023**	145	75 (67-82, 109)	19 (13-27, 28)	6 (2-11, 8)	0 (0-3, 0)	0 (0-3, 0)
**Jul 2023**	146	73 (65-80, 107)	21 (15-29, 31)	5 (2-11, 8)	0 (0-2, 0)	0 (0-2, 0)
**Aug 2023**	147	73 (65-80, 107)	22 (15-29, 32)	5 (2-10, 8)	0 (0-2, 0)	0 (0-2, 0)
**Sep 2023**	147	73 (65-80, 107)	22 (15-29, 32)	5 (2-10, 8)	0 (0-2, 0)	0 (0-2, 0)

b) NCRD

Data Cut	Total Cancers Considered	Concordant % (95% CI, n)	Discordant: SYMPLIFY stage > registry stage % (95% CI, n)	Discordant: Registry stage > SYMPLIFY stage % (95% CI, n)	Discordant: SYMPLIFY stage is "uncertain" % (95% CI, n)	Discordant: Registry stage is "uncertain" % (95% CI, n)
**Nov 2022**	230	73 (67-79, 169)	12 (8-17, 28)	9 (6-14, 21)	3 (1-6, 6)	3 (1-6, 6)
**Dec 2022**	231	74 (67-79, 170)	12 (9-17, 28)	9 (6-14, 21)	3 (1-6, 6)	3 (1-6, 6)
**Jan 2023**	230	74 (68-79, 170)	12 (8-17, 27)	9 (6-14, 21)	3 (1-6, 6)	3 (1-6, 6)
**Feb 2023**	234	73 (67-79, 171)	12 (8-16, 27)	9 (6-14, 22)	3 (1-6, 7)	3 (1-6, 7)
**Mar 2023**	236	73 (67-78, 172)	11 (8-16, 27)	9 (6-14, 22)	3 (1-7, 8)	3 (1-6, 7)
**Apr 2023**	236	73 (67-78, 172)	11 (8-16, 27)	9 (6-14, 22)	3 (1-7, 8)	3 (1-6, 7)
**May 2023**	-	-	-	-	-	-
**Jun 2023**	236	73 (67-78, 172)	12 (8-17, 28)	9 (6-14, 22)	3 (1-7, 8)	3 (1-5, 6)
**Jul 2023**	236	73 (67-78, 172)	12 (8-17, 28)	9 (6-14, 22)	3 (1-7, 8)	3 (1-5, 6)
**Aug 2023**	235	73 (67-78, 171)	12 (8-17, 28)	9 (6-14, 22)	3 (1-7, 8)	3 (1-5, 6)
**Sep 2023**	235	73 (67-78, 171)	12 (8-17, 28)	9 (6-14, 22)	3 (1-7, 8)	3 (1-5, 6)

c) DHCW and WCISU

Data Cut	Total Cancers Considered	Concordant % (95% CI, n)	Discordant: SYMPLIFY stage > registry stage % (95% CI, n)	Discordant: Registry stage > SYMPLIFY stage % (95% CI, n)	Discordant: SYMPLIFY stage is "uncertain" % (95% CI, n)	Discordant: Registry stage is "uncertain" % (95% CI, n)
**Sep 2022**	33	82 (65-93, 27)	9 (2-24, 3)	3 (0-16, 1)	6 (1-20, 2)	0 (0-10, 0)
**Oct 2022**	41	85 (71-94, 35)	12 (4-26, 5)	0 (0-8, 0)	2 (0-13, 1)	0 (0-8, 0)
**Nov 2022**	46	87 (74-95, 40)	11 (4-24, 5)	0 (0-8, 0)	2 (0-12, 1)	2 (0-12, 1)
**Dec 2022**	46	87 (74-95, 40)	11 (4-24, 5)	0 (0-8, 0)	2 (0-12, 1)	2 (0-12, 1)
**Jan 2023**	-	-	-	-	-	-
**Feb 2023**	46	87 (74-95, 40)	11 (4-24, 5)	0 (0-8, 0)	2 (0-12, 1)	2 (0-12, 1)
**Mar 2023**	46	87 (74-95, 40)	11 (4-24, 5)	0 (0-8, 0)	2 (0-12, 1)	2 (0-12, 1)
**Apr 2023**	-	-	-	-	-	-
**May 2023**	46	87 (74-95, 40)	11 (4-24, 5)	0 (0-8, 0)	2 (0-12, 1)	2 (0-12, 1)
**Jun 2023**	-	-	-	-	-	-
**Jul 2023**	98	83 (74-90, 81)	9.2 (9)	4.1 (4)	2.0 (2)	2.0 (2)

Table S10. Discordant ICD-10 cases based on corresponding cancer site groupings between SYMPLIFY and (a) RCRD, (b) NCRD, (c) DHCW, and (d) WCISU datasets at the final timepoint available for each dataset.

a)

RCRD Cancer Site – Sep 2023	SYMPLIFY Cancer Site – Jan 2023	Frequency
Oesophagus	Stomach	2
Uterus	Ovarian	1
Lymphoid	Colorectal	1
Colorectal	Anus	1
Pancreas	Colorectal	1
Colorectal	Stomach	1
Liver, bile duct	Lung, trachea, and bronchus	1
Ovarian	Uterus	1

b)

NCRD Cancer Site – Sep 2023	SYMPLIFY Cancer Site - Jan 2023	Frequency
Oesophagus	Stomach	3
Colorectal	Anus	1
Colorectal	Prostate	1
Liver, bile duct	Unknown primary	1
Lymphoid	Colorectal	1
Lymphoid	Unknown code	1
Other	Lymphoid	1
Uterus	Ovarian	1

c)

DHCW Cancer Site – May 2023	SYMPLIFY Cancer Site - Jan 2023	Frequency
Unknown primary	Bone and soft tissue (males), Ovarian (females)	2
Melanoma of skin	Colorectal	1
Uterus	Ovarian	1

d)

WCISU Cancer Site - Jul 2023	SYMPLIFY Cancer Site - Jan 2023	Frequency
Anus	Colorectal	8
Stomach	Lymphoid	1
Stomach	Oesophagus	1
Unknown primary	Lymphoid	1

Table S11. Discordant ICD-O-3 cases based on corresponding morphology groupings between SYMPLIFY and (a) RCRD, (b) NCRD, and (c) DHCW, and (d) WCISU datasets at the final timepoint available for comparison for each dataset.

a)

RCRD Morphology – Sep 2023	SYMPLIFY Morphology – Jan 2023	Frequency
Epithelial neoplasms, NOS	Adenomas and adenocarcinomas	5
Adenomas and adenocarcinomas	Epithelial neoplasms, NOS	4
Adenomas and adenocarcinomas	Cystic, mucinous, and serous neoplasms	3
Epithelial neoplasms, NOS	Ductal and lobular neoplasms	3
Cystic, mucinous, and serous neoplasms	Adenomas and adenocarcinomas	2
Myomatous neoplasms	Complex mixed and stromal neoplasms	2
Squamous cell neoplasms	Epithelial neoplasms, NOS	2
Blood vessel tumours	Ductal and lobular neoplasms	1
Blood vessel tumours	Epithelial neoplasms, NOS	1
Complex epithelial neoplasms	Epithelial neoplasms, NOS	1
Epithelial neoplasms, NOS	Cystic, mucinous, and serous neoplasms	1
Epithelial neoplasms, NOS	Squamous cell neoplasms	1
Lymphoid leukemias	Adenomas and adenocarcinomas	1
Malignant lymphomas, NOS or diffuse	Mature B-cell lymphomas	1
Neoplasms, NOS	Adenomas and adenocarcinomas	1
Neoplasms, NOS	Epithelial neoplasms, NOS	1

b)

NCRD Morphology – Sep 2023	SYMPLIFY Morphology - Jan 2023	Frequency
Cystic, mucinous, and serous neoplasms	Adenomas and adenocarcinomas	12
Adenomas and adenocarcinomas	Epithelial neoplasms, NOS	7
Acinar cell neoplasms	Adenomas and adenocarcinomas	3
Adenomas and adenocarcinomas	Cystic, mucinous, and serous neoplasms	3
Neoplasms, NOS	Adenomas and adenocarcinomas	3
Epithelial neoplasms, NOS	Adenomas and adenocarcinomas	2
Squamous cell neoplasms	Epithelial neoplasms, NOS	2
Acinar cell neoplasms	Squamous cell neoplasms	1
Complex epithelial neoplasms	Epithelial neoplasms, NOS	1
Complex epithelial neoplasms	Squamous cell neoplasms	1
Immunoproliferative diseases	Mature B-cell lymphomas	1
Lymphoid leukemias	Adenomas and adenocarcinomas	1
Myomatous neoplasms	Complex epithelial neoplasms	1
Neoplasms, NOS	Epithelial neoplasms, NOS	1

c)

DHCW Morphology - May 2023	SYMPLIFY Morphology - Jan 2023	Frequency
Adenomas and adenocarcinomas	Epithelial neoplasms, NOS	9
Squamous cell neoplasms	Epithelial neoplasms, NOS	3
Epithelial neoplasms, NOS	Squamous cell neoplasms	2
Cystic, mucinous, and serous neoplasms	Adenomas and adenocarcinomas	1

d)

WCISU Morphology - Jul 2023	SYMPLIFY Morphology - Jan 2023	Frequency
Adenomas and adenocarcinomas	Epithelial neoplasms, NOS	8
Squamous cell neoplasms	Epithelial neoplasms, NOS	3
Acinar cell neoplasms	Epithelial neoplasms, NOS	2
Cystic, mucinous, and serous neoplasms	Adenomas and adenocarcinomas	2
Neoplasms, NOS	Epithelial neoplasms, NOS	2
Acinar cell neoplasms	Adenomas and adenocarcinomas	1
Neoplasms, NOS	Adenomas and adenocarcinomas	1
Acinar cell neoplasms	Squamous cell neoplasms	1

Table S12. ICD-O-3 broad morphology groupings.

ICD-O-3 Code	Morphology	Broad Morphology Grouping
8000	Neoplasm	Neoplasms, NOS
8001	Tumour cells	Neoplasms, NOS
8002	Malignant tumour, small cell type	Neoplasms, NOS
8003	Malignant tumour, giant cell type	Neoplasms, NOS
8004	Malignant tumour, spindle cell type	Neoplasms, NOS
8005	Malignant tumour, clear cell type	Neoplasms, NOS
8010	Carcinoma, NOS	Epithelial neoplasms, NOS
8011	Epithelioma, malignant	Epithelial neoplasms, NOS
8012	Large cell carcinoma, NOS	Epithelial neoplasms, NOS
8013	Large call neuroendocrine carcinoma	Epithelial neoplasms, NOS
8014	Large cell carcinoma with rhabdoid phenotype	Epithelial neoplasms, NOS
8015	Glassy cell carcinoma	Epithelial neoplasms, NOS
8020	Carcinoma, undifferentiated, NOS	Epithelial neoplasms, NOS
8021	Carcinoma, anaplastic, NOS	Epithelial neoplasms, NOS
8022	Pleomorphic carcinoma	Epithelial neoplasms, NOS
8030	Giant cell and spindle cell carcinoma	Epithelial neoplasms, NOS
8031	Giant cell carcinoma	Epithelial neoplasms, NOS
8032	Spindle cell carcinoma, NOS	Epithelial neoplasms, NOS
8033	Pseudosarcomatous carcinoma	Epithelial neoplasms, NOS
8034	Polygonal cell carcinoma	Epithelial neoplasms, NOS
8035	Carcinoma with osteoclast-like giant cells	Epithelial neoplasms, NOS
8041	Small cell carcinoma, NOS	Epithelial neoplasms, NOS
8042	Oat cell carcinoma	Epithelial neoplasms, NOS

8043	Small cell carcinoma, fusiform cell	Epithelial neoplasms, NOS
8044	Small cell carcinoma, intermediate cell	Epithelial neoplasms, NOS
8045	Combined small cell carcinoma	Epithelial neoplasms, NOS
8046	Non-small cell carcinoma	Epithelial neoplasms, NOS
8050	Papillary carcinoma, NOS	Squamous cell neoplasms
8051	Verrucous carcinoma, NOS	Squamous cell neoplasms
8052	Papillary squamous cell carcinoma	Squamous cell neoplasms
8070	Squamous cell carcinoma, NOS	Squamous cell neoplasms
8071	Squamous cell carcinoma, keratinizing, NOS	Squamous cell neoplasms
8072	Squamous cell carcinoma, large cell, nonkeratinizing, NOS	Squamous cell neoplasms
8073	Squamous cell carcinoma, small cell, nonkeratinizing	Squamous cell neoplasms
8074	Squamous cell carcinoma, spindle cell	Squamous cell neoplasms
8075	Squamous cell carcinoma, adenoid	Squamous cell neoplasms
8076	Squamous cell carcinoma, microinvasive	Squamous cell neoplasms
8078	Squamous cell carcinoma with horn formation	Squamous cell neoplasms
8082	Lymphoepithelial carcinoma	Squamous cell neoplasms
8083	Basaloid squamous cell carcinoma	Squamous cell neoplasms
8084	Squamous cell carcinoma, clear cell type	Squamous cell neoplasms
8090	Basal cell carcinoma, NOS (C44._)	Basal cell neoplasms
8091	Multifocal superficial basal cell carcinoma (C44._)	Basal cell neoplasms
8092	Infiltrating basal cell carcinoma, NOS (C44._)	Basal cell neoplasms
8093	Basal cell carcinoma, fibroepithelial (C44._)	Basal cell neoplasms
8094	Basosquamous carcinoma (C44._)	Basal cell neoplasms
8095	Metatypical carcinoma (C44._)	Basal cell neoplasms
8097	Basal cell carcinoma, nodular (C44._)	Basal cell neoplasms
8098	Adenoid basal carcinoma (C53._)	Basal cell neoplasms
8102	Trichilemmocarcinoma (C44._)	Basal cell neoplasms
8110	Pilomatrix carcinoma (C44._)	Basal cell neoplasms

8120	Transitional cell carcinoma, NOS	Transitional cell papillomas and carcinomas
8121	Schneiderian carcinoma (C30.0, C31._)	Transitional cell papillomas and carcinomas
8122	Transitional cell carcinoma, spindle cell	Transitional cell papillomas and carcinomas
8123	Basaloid carcinoma	Transitional cell papillomas and carcinomas
8124	Cloacogenic carcinoma (C21.2)	Transitional cell papillomas and carcinomas
8130	Papillary transitional cell carcinoma (C67._)	Transitional cell papillomas and carcinomas
8131	Transitional cell carcinoma, micropapillary (C67._)	Transitional cell papillomas and carcinomas
8140	Adenocarcinoma, NOS	Adenomas and adenocarcinomas
8141	Scirrhous adenocarcinoma	Adenomas and adenocarcinomas
8142	Linitis plastica (C16._)	Adenomas and adenocarcinomas
8143	Superficial spreading adenocarcinoma	Adenomas and adenocarcinomas
8144	Adenocarcinoma, intestinal type (C16._)	Adenomas and adenocarcinomas
8145	Carcinoma, diffuse type (C16._)	Adenomas and adenocarcinomas
8147	Vasal cell adenocarcinoma	Adenomas and adenocarcinomas
8150	Pancreatic endocrine tumour (C25._)	Adenomas and adenocarcinomas
8151	Insulinoma, malignant (C25._)	Adenomas and adenocarcinomas
8152	Glucagonoma, malignant (C25._)	Adenomas and adenocarcinomas
8153	Gastrinoma, malignant	Adenomas and adenocarcinomas
8154	Mixed pancreatic endocrine and exocrine tumour, malignant (C25._)	Adenomas and adenocarcinomas
8155	Vipoma	Adenomas and adenocarcinomas
8156	Somatostatinoma	Adenomas and adenocarcinomas
8160	Cholangiocarcinoma (C22.1, C24.0)	Adenomas and adenocarcinomas
8161	Bile duct cystadenocarcinoma (C22.1, C24.0)	Adenomas and adenocarcinomas
8162	Klatskin tumour (C22.1, C24.0)	Adenomas and adenocarcinomas
8163	Pancreatobiliary-type carcinoma (C24.1)	Adenomas and adenocarcinomas
8170	Hepatocellular carcinoma, NOS (C22.0)	Adenomas and adenocarcinomas
8171	Hepatocellular carcinoma, fibrolamellar (C22.0)	Adenomas and adenocarcinomas
8172	Hepatocellular carcinoma, scirrhous (C22.0)	Adenomas and adenocarcinomas

8173	Hepatocellular carcinoma, spindle cell variant (C22.0)	Adenomas and adenocarcinomas
8174	Hepatocellular carcinoma, clear cell type (C22.0)	Adenomas and adenocarcinomas
8175	Hepatocellular carcinoma, pleomorphic type (C22.0)	Adenomas and adenocarcinomas
8180	Combined hepatocellular carcinoma and cholangiocarcinoma (C22.0)	Adenomas and adenocarcinomas
8190	Trabecular adenocarcinoma	Adenomas and adenocarcinomas
8200	Adenoid cystic carcinoma	Adenomas and adenocarcinomas
8201	Cribriform carcinoma, NOS	Adenomas and adenocarcinomas
8210	Adenocarcinoma in adenomatous polyp	Adenomas and adenocarcinomas
8211	Tubular adenocarcinoma	Adenomas and adenocarcinomas
8213	Serrated adenocarcinoma	Adenomas and adenocarcinomas
8214	Parietal cell carcinoma (C16._)	Adenomas and adenocarcinomas
8215	Adenocarcinoma of anal glands (C21.1)	Adenomas and adenocarcinomas
8220	Adenocarcinoma in adenomatous polyposis coli (C18._)	Adenomas and adenocarcinomas
8221	Adenocarcinoma in multiple adenomatous polyps (C18._)	Adenomas and adenocarcinomas
8230	Solid carcinoma, NOS	Adenomas and adenocarcinomas
8231	Carcinoma simplex	Adenomas and adenocarcinomas
8240	Carcinoid tumour, NOS	Adenomas and adenocarcinomas
8241	Enterochromaffin cell carcinoid	Adenomas and adenocarcinomas
8242	Enterochromaffin-like cell tumour	Adenomas and adenocarcinomas
8243	Goblet cell carcinoid	Adenomas and adenocarcinomas
8244	Mixed adenoneuroendocrine carcinoma	Adenomas and adenocarcinomas
8245	Adenocarcinoid tumour	Adenomas and adenocarcinomas
8246	Neuroendocrine carcinoma, NOS	Adenomas and adenocarcinomas
8247	Merkel cell carcinoma (C44._)	Adenomas and adenocarcinomas
8249	Atypical carcinoid tumour	Adenomas and adenocarcinomas
8250	Bronchiolo-alveolar adenocarcinoma, NOS	Adenomas and adenocarcinomas
8251	Alveolar adenocarcinoma (C34._)	Adenomas and adenocarcinomas
8252	Bronchiolo-alveolar carcinoma, non-mucinous (C34._)	Adenomas and adenocarcinomas
8253	Bronchiolo-alveolar carcinoma, mucinous (C34._)	Adenomas and adenocarcinomas

8254	Bronchiolo-alveolar carcinoma, mixed mucinous and non-mucinous (C34._)	Adenomas and adenocarcinomas
8255	Adenocarcinoma with mixed subtypes	Adenomas and adenocarcinomas
8260	Papillary adenocarcinoma, NOS	Adenomas and adenocarcinomas
8261	Adenocarcinoma in villous adenoma	Adenomas and adenocarcinomas
8262	Villous adenocarcinoma	Adenomas and adenocarcinomas
8263	Adenocarcinoma in tubulovillous adenoma	Adenomas and adenocarcinomas
8265	Micropapillary carcinoma, NOS (C18._, C19.9, C20.9)	Adenomas and adenocarcinomas
8270	Chromophobe carcinoma (C75.1)	Adenomas and adenocarcinomas
8272	Pituitary carcinoma, NOS (C75.1)	Adenomas and adenocarcinomas
8280	Acidophil carcinoma (C75.1)	Adenomas and adenocarcinomas
8281	Mixed acidophil-basophil carcinoma (C75.1)	Adenomas and adenocarcinomas
8290	Oxyphilic adenocarcinoma	Adenomas and adenocarcinomas
8300	Basophil carcinoma (C75.1)	Adenomas and adenocarcinomas
8310	Clear cell adenocarcinoma, NOS	Adenomas and adenocarcinomas
8312	Renal cell carcinoma, NOS (C64.9)	Adenomas and adenocarcinomas
8313	Clear cell adenocarcinofibroma (C56.9)	Adenomas and adenocarcinomas
8314	Lipid-rich carcinoma (C50._)	Adenomas and adenocarcinomas
8315	Glycogen-rich carcinoma	Adenomas and adenocarcinomas
8316	Cyst-associated renal cell carcinoma (C64.9)	Adenomas and adenocarcinomas
8317	Renal cell carcinoma, chromophobe type (C64.9)	Adenomas and adenocarcinomas
8318	Renal cell carcinoma, sarcomatoid (C64.9)	Adenomas and adenocarcinomas
8319	Collecting duct carcinoma (C64.9)	Adenomas and adenocarcinomas
8320	Glandular cell carcinoma	Adenomas and adenocarcinomas
8322	Water-clear cell adenocarcinoma (C75.0)	Adenomas and adenocarcinomas
8323	Mixed cell adenocarcinoma	Adenomas and adenocarcinomas
8330	Follicular adenocarcinoma, NOS (C73.9)	Adenomas and adenocarcinomas
8331	Follicular adenocarcinoma, well differentiated (C73.9)	Adenomas and adenocarcinomas
8332	Follicular adenocarcinoma, trabecular (C73.9)	Adenomas and adenocarcinomas
8333	Fetal adenocarcinoma	Adenomas and adenocarcinomas

8335	Follicular carcinoma, minimally invasive (C73.9)	Adenomas and adenocarcinomas
8337	Insular carcinoma (C73.9)	Adenomas and adenocarcinomas
8340	Papillary carcinoma, follicular variant (C73.9)	Adenomas and adenocarcinomas
8341	Papillary microcarcinoma (C73.9)	Adenomas and adenocarcinomas
8342	Papillary carcinoma, oxyphilic cell (C73.9)	Adenomas and adenocarcinomas
8343	Papillary carcinoma, encapsulated (C73.9)	Adenomas and adenocarcinomas
8344	Papillary carcinoma, columnar cell (C73.9)	Adenomas and adenocarcinomas
8345	Medullary carcinoma with amyloid stroma (C73.9)	Adenomas and adenocarcinomas
8346	Mixed medullary-follicular carcinoma (C73.9)	Adenomas and adenocarcinomas
8347	Mixed medullary-papillary carcinoma (C73.9)	Adenomas and adenocarcinomas
8350	Nonencapsulated sclerosing carcinoma (C73.9)	Adenomas and adenocarcinomas
8370	Adrenal cortical carcinoma (C74.0)	Adenomas and adenocarcinomas
8380	Endometrioid adenocarcinoma, NOS	Adenomas and adenocarcinomas
8381	Endometrioid adenofibroma, malignant	Adenomas and adenocarcinomas
8382	Endometrioid adenocarcinoma, secretory variant	Adenomas and adenocarcinomas
8383	Endometrioid adenocarcinoma, ciliated cell variant	Adenomas and adenocarcinomas
8384	Adenocarcinoma, endocervical type	Adenomas and adenocarcinomas
8390	Skin appendage carcinoma (C44._)	Adnexal and skin appendage neoplasms
8400	Sweat gland adenocarcinoma (C44._)	Adnexal and skin appendage neoplasms
8401	Apocrine adenocarcinoma (C44._)	Adnexal and skin appendage neoplasms
8402	Nodular hidradenoma, malignant (C44._)	Adnexal and skin appendage neoplasms
8403	Malignant eccrine spiradenoma (C44._)	Adnexal and skin appendage neoplasms
8407	Sclerosing sweat duct carcinoma (C44._)	Adnexal and skin appendage neoplasms
8408	Eccrine papillary adenocarcinoma (C44._)	Adnexal and skin appendage neoplasms
8409	Eccrine poroma, malignant	Adnexal and skin appendage neoplasms
8410	Sebaceous adenocarcinoma (C44._)	Adnexal and skin appendage neoplasms
8413	Eccrine adenocarcinoma (C44._)	Adnexal and skin appendage neoplasms
8420	Ceruminous adenocarcinoma (C44.2)	Adnexal and skin appendage neoplasms

8430	Mucoepidermoid carcinoma	Mucoepidermoid neoplasms
8440	Cystadenocarcinoma	Cystic, mucinous, and serous neoplasms
8441	Serous cystadenocarcinoma, NOS (C56.9)	Cystic, mucinous, and serous neoplasms
8450	Papillary cystadenocarcinoma, NOS (C56.9)	Cystic, mucinous, and serous neoplasms
8452	Solid pseudopapillary carcinoma (C25._)	Cystic, mucinous, and serous neoplasms
8453	Intraductal papillary mucinous carcinoma, invasive (C25._)	Cystic, mucinous, and serous neoplasms
8460	Papillary serous cystadenocarcinoma (C56.9)	Cystic, mucinous, and serous neoplasms
8461	Serous surface papillary carcinoma (C56.9)	Cystic, mucinous, and serous neoplasms
8470	Mucinous cystadenocarcinoma, NOS (C56.9)	Cystic, mucinous, and serous neoplasms
8471	Papillary mucinous cystadenocarcinoma, NOS (C56.9)	Cystic, mucinous, and serous neoplasms
8480	Mucinous adenocarcinoma	Cystic, mucinous, and serous neoplasms
8481	Mucin-producing adenocarcinoma	Cystic, mucinous, and serous neoplasms
8482	Mucinous adenocarcinoma endocervical type	Cystic, mucinous, and serous neoplasms
8490	Signet ring cell carcinoma	Cystic, mucinous, and serous neoplasms
8500	Infiltrating duct carcinoma, NOS (C50._)	Ductal and lobular neoplasms
8501	Comedocarcinoma, NOS (C50._)	Ductal and lobular neoplasms
8502	Secretory carcinoma of breast (C50._)	Ductal and lobular neoplasms
8503	Intraductal papillary adenocarcinoma with invasion (C50._)	Ductal and lobular neoplasms
8504	Intracystic carcinoma, NOS	Ductal and lobular neoplasms
8508	Cystic hypersecretory carcinoma (C50._)	Ductal and lobular neoplasms
8510	Medullary carcinoma, NOS	Ductal and lobular neoplasms
8512	Medullary carcinoma with lymphoid stroma	Ductal and lobular neoplasms
8513	Atypical medullary carcinoma (C50._)	Ductal and lobular neoplasms
8514	Duct carcinoma, desmoplastic type	Ductal and lobular neoplasms
8520	Lobular carcinoma, NOS (C50._)	Ductal and lobular neoplasms
8521	Infiltrating ductular carcinoma (C50._)	Ductal and lobular neoplasms
8522	Infiltrating duct and lobular carcinoma (C50._)	Ductal and lobular neoplasms
8523	Infiltrating duct mixed with other types of carcinoma (C50._)	Ductal and lobular neoplasms

8524	Infiltrating lobular mixed with other types of carcinoma (C50._)	Ductal and lobular neoplasms
8525	Polymorphous low grade adenocarcinoma	Ductal and lobular neoplasms
8530	Inflammatory carcinoma (C50._)	Ductal and lobular neoplasms
8540	Paget disease, mammary(C50._)	Ductal and lobular neoplasms
8541	Paget disease, and infiltrating duct carcinoma of breast (C50._)	Ductal and lobular neoplasms
8542	Paget disease, extramammary (except Paget disease of bone)	Ductal and lobular neoplasms
8543	Paget disease and intraductal carcinoma of breast (C50._)	Ductal and lobular neoplasms
8550	Acinar cell carcinoma	Acinar cell neoplasms
8551	Acinar cell cystadenocarcinoma	Acinar cell neoplasms
8552	Mixed acinar-ductal carcinoma	Acinar cell neoplasms
8560	Adenosquamous carcinoma	Complex epithelial neoplasms
8562	Epithelial-myoepithelial carcinoma	Complex epithelial neoplasms
8570	Adenocarcinoma with squamous metaplasia	Complex epithelial neoplasms
8571	Adenocarcinoma with cartilaginous and osseous metaplasia	Complex epithelial neoplasms
8572	Adenocarcinoma with spindle cell metaplasia	Complex epithelial neoplasms
8573	Adenocarcinoma with apocrine metaplasia	Complex epithelial neoplasms
8574	Adenocarcinoma with neuroendocrine differentiation	Complex epithelial neoplasms
8575	Metaplastic carcinoma, NOS	Complex epithelial neoplasms
8576	Hepatoid adenocarcinoma	Complex epithelial neoplasms
8580	Thymoma, malignant, NOS (C37.9)	Thymic epithelial neoplasms
8581	Thymoma, type A, malignant (C37.9)	Thymic epithelial neoplasms
8582	Thymoma, type AB, malignant (C37.9)	Thymic epithelial neoplasms
8583	Thymoma, type B1, malignant (C37.9)	Thymic epithelial neoplasms
8584	Thymoma, type B2, malignant (C37.9)	Thymic epithelial neoplasms
8585	Thymoma, type B3, malignant (C37.9)	Thymic epithelial neoplasms
8586	Thymic carcinoma, NOS (C37.9)	Thymic epithelial neoplasms
8588	Spindle epithelial tumour with thymus-like element	Thymic epithelial neoplasms
8589	Carcinoma showing thymus-like element	Thymic epithelial neoplasms

8600	Thecoma, malignant (C56.9)	Specialized gonadal neoplasms
8620	Granulosa cell tumour, malignant (C56.9)	Specialized gonadal neoplasms
8630	Androblastoma, malignant	Specialized gonadal neoplasms
8631	Sertoli-Leydig cell tumour, poorly differentiated	Specialized gonadal neoplasms
8634	Sertoli-Leydig cell tumour, poorly differentiated, with heterologous elements	Specialized gonadal neoplasms
8640	Sertoli cell carcinoma (C62._)	Specialized gonadal neoplasms
8650	Leydig cell tumour, malignant (C62._)	Specialized gonadal neoplasms
8670	Steroid cell tumour, malignant	Specialized gonadal neoplasms
8680	Paraganglioma, malignant	Paragangliomas and glomus tumours
8693	Extra-adrenal paraganglioma, malignant	Paragangliomas and glomus tumours
8700	Pheochromocytoma, malignant (C74.1)	Paragangliomas and glomus tumours
8710	Glomangiosarcoma	Paragangliomas and glomus tumours
8711	Glomus tumour, malignant	Paragangliomas and glomus tumours
8720	Malignant melanoma, NOS	Nevi and melanomas
8721	Nodular melanoma (C44._)	Nevi and melanomas
8722	Balloon cell melanoma (C44._)	Nevi and melanomas
8723	Malignant melanoma, regressing (C44._)	Nevi and melanomas
8728	Meningeal melanomatosis (C70.9)	Nevi and melanomas
8730	Amelanotic melanoma (C44._)	Nevi and melanomas
8740	Malignant melanoma in junctional nevus (C44._)	Nevi and melanomas
8741	Malignant melanoma in precancerous melanosis (C44._)	Nevi and melanomas
8742	Lentigo maligna melanoma (C44._)	Nevi and melanomas
8743	Superficial spreading melanoma (C44._)	Nevi and melanomas
8744	Acral lentiginous melanoma, malignant (C44._)	Nevi and melanomas
8745	Desmoplastic melanoma malignant (C44._)	Nevi and melanomas
8746	Mucosal lentiginous melanoma	Nevi and melanomas
8761	Malignant melanoma in giant pigmented nevus (C44._)	Nevi and melanomas
8770	Mixed epithelioid and spindle cell melanoma	Nevi and melanomas

8771	Epithelioid cell melanoma	Nevi and melanomas
8772	Spindle cell melanoma, NOS	Nevi and melanomas
8773	Spindle cell melanoma, type A (C69._)	Nevi and melanomas
8774	Spindle cell melanoma, type B (C69._)	Nevi and melanomas
8780	Blue nevus, malignant (C44._)	Nevi and melanomas
8800	Sarcoma, NOS	Nevi and melanomas
8801	Spindle cell sarcoma	Nevi and melanomas
8802	Giant cell sarcoma	Nevi and melanomas
8803	Small cell sarcoma	Nevi and melanomas
8804	Epithelioid sarcoma	Nevi and melanomas
8805	Undifferentiated sarcoma	Nevi and melanomas
8806	Desmoplastic small round cell tumour	Nevi and melanomas
8810	Fibrosarcoma, NOS	Fibromatous neoplasms
8811	Fibromyxosarcoma	Fibromatous neoplasms
8812	Periosteal fibrosarcoma (C40._, C41._)	Fibromatous neoplasms
8813	Fascial fibrosarcoma	Fibromatous neoplasms
8814	Infantile fibrosarcoma	Fibromatous neoplasms
8815	Solitary fibrous tumour, malignant	Fibromatous neoplasms
8830	Malignant fibrous histiocytoma	Fibromatous neoplasms
8832	Dermatofibrosarcoma, NOS (C44._)	Fibromatous neoplasms
8833	Pigmented dermatofibrosarcoma protuberans (C44._)	Fibromatous neoplasms
8840	Myxosarcoma	Myxomatous neoplasms
8850	Liposarcoma, NOS	Lipomatous neoplasms
8851	Liposarcoma, well-differentiated	Lipomatous neoplasms
8852	Myxoid liposarcoma	Lipomatous neoplasms
8853	Round cell liposarcoma	Lipomatous neoplasms
8854	Pleomorphic liposarcoma	Lipomatous neoplasms
8855	Mixed liposarcoma	Lipomatous neoplasms

8857	Fibroblastic liposarcoma	Lipomatous neoplasms
8858	Dedifferentiated liposarcoma	Lipomatous neoplasms
8890	Leiomymosarcoma, NOS	Myomatous neoplasms
8891	Epithelioid leiomyosarcoma	Myomatous neoplasms
8894	Angiomyosarcoma	Myomatous neoplasms
8895	Myosarcoma	Myomatous neoplasms
8896	Myxoid leiomyosarcoma	Myomatous neoplasms
8900	Rhabdomyosarcoma, NOS	Myomatous neoplasms
8901	Pleomorphic rhabdomyosarcoma, adult type	Myomatous neoplasms
8902	Mixed type rhabdomyosarcoma	Myomatous neoplasms
8910	Embryonal rhabdomyosarcoma, NOS	Myomatous neoplasms
8912	Spindle cell rhabdomyosarcoma	Myomatous neoplasms
8920	Alveolar rhabdomyosarcoma	Myomatous neoplasms
8921	Rhabdomyosarcoma with ganglionic differentiation	Myomatous neoplasms
8930	Endometrial stromal sarcoma, NOS (C54.1)	Complex mixed and stromal neoplasms
8931	Endometrial stromal sarcoma, low grade (C54.1)	Complex mixed and stromal neoplasms
8933	Adenosarcoma	Complex mixed and stromal neoplasms
8934	Carcinofibroma	Complex mixed and stromal neoplasms
8935	Stromal sarcoma, NOS	Complex mixed and stromal neoplasms
8936	Gastrointestinal stromal sarcoma	Complex mixed and stromal neoplasms
8940	Mixed tumour, malignant, NOS	Complex mixed and stromal neoplasms
8941	Carcinoma in pleomorphic adenoma (C07._, C08._)	Complex mixed and stromal neoplasms
8950	Mullerian mixed tumour (C54._)	Complex mixed and stromal neoplasms
8951	Mesodermal mixed tumour	Complex mixed and stromal neoplasms
8959	Malignant cystic nephroma (C64.9)	Complex mixed and stromal neoplasms
8960	Nephroblastoma, NOS	Complex mixed and stromal neoplasms
8963	Malignant rhabdoid tumour	Complex mixed and stromal neoplasms
8964	Clear cell sarcoma of kidney (C64.9)	Complex mixed and stromal neoplasms

8970	Hepatoblastoma (C22.0)	Complex mixed and stromal neoplasms
8971	Pancreatoblastoma (C25._)	Complex mixed and stromal neoplasms
8972	Pulmonary blastoma (C34._)	Complex mixed and stromal neoplasms
8973	Pleuropulmonary blastoma	Complex mixed and stromal neoplasms
8980	Carcinosarcoma, NOS	Complex mixed and stromal neoplasms
8981	Carcinosarcoma, embryonal	Complex mixed and stromal neoplasms
8982	Malignant myoepithelioma	Complex mixed and stromal neoplasms
8990	Mesenchymoma, malignant	Complex mixed and stromal neoplasms
8991	Embryonal sarcoma	Complex mixed and stromal neoplasms
9000	Brenner tumour, malignant (C56.9)	Fibroeptihelial neoplasms
9014	Serous adenocarcinofibroma	Fibroeptihelial neoplasms
9015	Mucinous adenocarcinofibroma	Fibroeptihelial neoplasms
9020	Phyllodes tumour, malignant (C50._)	Fibroeptihelial neoplasms
9040	Synovial sarcoma, NOS	Synovial-like neoplasms
9041	Synovial sarcoma, spindle cell	Synovial-like neoplasms
9042	Synovial sarcoma, epithelioid cell	Synovial-like neoplasms
9043	Synovial sarcoma, biphasic	Synovial-like neoplasms
9044	Clear cell sarcoma, NOS	Synovial-like neoplasms
9050	Mesothelioma, malignant	Mesothelial neoplasms
9051	Fibrous mesothelioma, malignant	Mesothelial neoplasms
9052	Epithelioid mesothelioma, malignant	Mesothelial neoplasms
9053	Mesothelioma, biphasic, malignant	Mesothelial neoplasms
9060	Dysgerminoma	Germ cell neoplasms
9061	Seminoma, NOS (C62._)	Germ cell neoplasms
9062	Seminoma, anaplastic (C62._)	Germ cell neoplasms
9063	Spermatocytic seminoma (C62._)	Germ cell neoplasms
9064	Germinoma	Germ cell neoplasms
9065	Germ cell tumour, nonseminomatous (C62._)	Germ cell neoplasms

9070	Embryonal carcinoma, NOS	Germ cell neoplasms
9071	Yolk sac tumour	Germ cell neoplasms
9072	Polyembroma	Germ cell neoplasms
9080	Teratoma, malignant, NOS	Germ cell neoplasms
9081	Teratocarcinoma	Germ cell neoplasms
9082	Malignant teratoma, undifferentiated	Germ cell neoplasms
9083	Malignant teratoma, intermediate	Germ cell neoplasms
9084	Teratoma with malignant transformation	Germ cell neoplasms
9085	Mixed germ cell tumour	Germ cell neoplasms
9090	Struma ovarii, malignant (C56.9)	Germ cell neoplasms
9100	Choriocarcinoma, NOS	Trophoblastic neoplasms
9101	Choriocarcinoma combined with other germ cell elements	Trophoblastic neoplasms
9102	Malignant teratoma, trophoblastic	Trophoblastic neoplasms
9105	Trophoblastic tumour, epithelioid	Trophoblastic neoplasms
9110	Mesonephroma, malignant	Mesonephromas
9120	Hemangiosarcoma	Blood vessel tumours
9124	Kupffer cell sarcoma (C22.0)	Blood vessel tumours
9130	Hemangioendothelioma, malignant	Blood vessel tumours
9133	Epithelioid hemangioendothelioma, malignant	Blood vessel tumours
9140	Kaposi sarcoma	Blood vessel tumours
9150	Hemangiopericytoma, malignant	Blood vessel tumours
9170	Lymphangiosarcoma	Lymphatic vessel tumours
9180	Osteosarcoma, NOS (C40._, C41._)	Osseous and chonrdromatous neoplasms
9181	Chondroblastic osteosarcoma (C40._, C41._)	Osseous and chonrdromatous neoplasms
9182	Fibroblastic osteosarcoma (C40._, C41._)	Osseous and chonrdromatous neoplasms
9183	Telangiectatic osteosarcoma (C40._, C41._)	Osseous and chonrdromatous neoplasms
9184	Osteosarcoma in Paget disease of bone (C40._, C41._)	Osseous and chonrdromatous neoplasms
9185	Small cell osteosarcoma (C40._, C41._)	Osseous and chonrdromatous neoplasms

9186	Central osteosarcoma (C40._, C41._)	Osseous and chonrdromatous neoplasms
9187	Intraosseous well differentiated osteosarcoma (C40._, C41._)	Osseous and chonrdromatous neoplasms
9192	Parosteal osteosarcoma (C40._, C41._)	Osseous and chonrdromatous neoplasms
9193	Periosteal osteosarcoma (C40._, C41._)	Osseous and chonrdromatous neoplasms
9194	High grade surface osteosarcoma (C40._, C41._)	Osseous and chonrdromatous neoplasms
9195	Intracortical osteosarcoma (C40._, C41._)	Osseous and chonrdromatous neoplasms
9220	Chondrosarcoma, NOS (C40._, C41._)	Osseous and chonrdromatous neoplasms
9221	Juxtacortical chondrosarcoma (C40._, C41._)	Osseous and chonrdromatous neoplasms
9230	Chrondroblastoma, malignant (C40._, C41._)	Osseous and chonrdromatous neoplasms
9231	Myxoid chrondrosarcoma	Osseous and chonrdromatous neoplasms
9240	Mesenchymal chondrosarcoma	Osseous and chonrdromatous neoplasms
9242	Clear cell chondrosarcoma (C40._, C41._)	Osseous and chonrdromatous neoplasms
9243	Dedifferentiated chondrosarcoma (C40._, C41._)	Osseous and chonrdromatous neoplasms
9250	Giant cell tumour of bone, malignant (C40._, C41._)	Giant cell tumours
9251	Malignant giant cell tumour of soft parts	Giant cell tumours
9252	Malignant tenosynovial giant cell tumour (C49._)	Giant cell tumours
9260	Ewing sarcoma	Miscellaneous bone tumours
9261	Adamantinoma of long bones (C40._)	Miscellaneous bone tumours
9270	Odontogenic tumour, malignant	Odontogenic tumours
9290	Ameloblastic odontosarcoma	Odontogenic tumours
9310	Ameloblastoma, malignant	Odontogenic tumours
9330	Ameloblastic fibrosarcoma	Odontogenic tumours
9342	Odontogenic carcinosarcoma	Odontogenic tumours
9362	Pineoblastoma (C75.3)	Miscellaneous tumours
9264	Peripheral neuroectodermal tumour	Miscellaneous tumours
9365	Askin tumour	Miscellaneous tumours
9370	Chordoma, NOS	Miscellaneous tumours
9371	Chondroid chordoma	Miscellaneous tumours

9372	Dedifferentiated chordoma	Miscellaneous tumours
9380	Glioma, malignant (C71._)	Gliomas
9381	Gliomatosis cerebri (C71._)	Gliomas
9382	Mixed glioma (C71._)	Gliomas
9390	Choroid plexus carcinoma (C71.5)	Gliomas
9391	Ependymoma, NOS (C71._)	Gliomas
9392	Ependymoma, anaplastic (C71._)	Gliomas
9393	Papillary ependymoma (C71._)	Gliomas
9395	Papillary tumour of the pineal region	Gliomas
9400	Astrocytoma, NOS	Gliomas
9401	Astrocytoma, anaplastic (C71._)	Gliomas
9410	Protoplasmic astrocytoma (C71._)	Gliomas
9411	Gemistocytic astrocytoma (C71._)	Gliomas
9420	Fibrillary astrocytoma (C71._)	Gliomas
9423	Polar spongioblastoma (C71._)	Gliomas
9424	Pleomorphic xanthoastrocytoma (C71._)	Gliomas
9425	Pilomyxoid astrocytoma	Gliomas
9430	Astroblastoma (C71._)	Gliomas
9440	Glioblastoma, NOS (C71._)	Gliomas
9441	Giant cell glioblastoma (C71._)	Gliomas
9442	Gliosarcoma (C71._)	Gliomas
9450	Oligodendroglioma, NOS (C71._)	Gliomas
9451	Oligodendroglioma, anaplastic (C71._)	Gliomas
9460	Oligodendroblastoma (C71._)	Gliomas
9470	Medulloblastoma, NOS (C71.6)	Gliomas
9471	Desmoplastic nodular medulloblastoma (C71.6)	Gliomas
9472	Medullomyoblastoma (C71.6)	Gliomas
9473	Primitive neuroectodermal tumour, NOS	Gliomas
9474	Large cell medulloblastoma (C71.6)	Gliomas

9480	Cerebellar sarcoma, NOS (C71.6)	Gliomas
9493	Dysplastic gangliocytoma of cerebellum	Neuroepitheliomatous neoplasms
9500	Neuroblastoma, NOS	Neuroepitheliomatous neoplasms
9501	Medulloepithelioma, NOS	Neuroepitheliomatous neoplasms
9502	Teratoid medulloepithelioma	Neuroepitheliomatous neoplasms
9503	Neuroepithelioma, NOS	Neuroepitheliomatous neoplasms
9504	Spongioneuroblastoma	Neuroepitheliomatous neoplasms
9505	Ganglioma, anaplastic	Neuroepitheliomatous neoplasms
9508	Atypical teratoid/rhabdoid tumour (C71._)	Neuroepitheliomatous neoplasms
9510	Retinoblastoma, NOS (C69.2)	Neuroepitheliomatous neoplasms
9511	Retinoblastoma, differentiated (C69.2)	Neuroepitheliomatous neoplasms
9512	Retinoblastoma, undifferentiated (C69.2)	Neuroepitheliomatous neoplasms
9513	Retinoblastoma, diffuse (C69.2)	Neuroepitheliomatous neoplasms
9520	Olfactory neurogenic tumour	Neuroepitheliomatous neoplasms
9521	Olfactory neurocytoma (C30.0)	Neuroepitheliomatous neoplasms
9522	Olfactory neuroblastoma (C30.0)	Neuroepitheliomatous neoplasms
9523	Olfactory neuroepithelioma (C30.0)	Neuroepitheliomatous neoplasms
9530	Meningioma, malignant	Meningiomas
9538	Papillary meningioma	Meningiomas
9539	Meningeal sarcomatosis	Meningiomas
9540	Malignant peripheral nerve sheath tumour	Nerve sheath tumours
9560	Neurilemoma, malignant	Nerve sheath tumours
9561	Malignant peripheral nerve sheath tumour with rhabdomyoblastic differentiation	Nerve sheath tumours
9571	Perineurioma, malignant	Nerve sheath tumours
9580	Granular cell tumour, malignant	Granular cell tumours and alveolar soft part sarcomas
9581	Alveolar soft part sarcoma	Granular cell tumours and alveolar soft part sarcomas
9590	Malignant lymphoma, NOS	Malignant lymphomas, NOS or diffuse
9591	Malignant lymphoma, non-Hodgkin, NOS	Malignant lymphomas, NOS or diffuse

9596	Composite Hodgkin and non-Hodgkin lymphoma	Malignant lymphomas, NOS or diffuse
9597	Primary cutaneous follicle centre lymphoma	Malignant lymphomas, NOS or diffuse
9650	Hodgkin lymphoma, NOS	Hodgkin lymphoma
9651	Hodgkin lymphoma, lymphocyte-rich	Hodgkin lymphoma
9652	Hodgkin lymphoma, mixed cellularity, NOS	Hodgkin lymphoma
9653	Hodgkin lymphoma, lymphocyte depletion, NOS	Hodgkin lymphoma
9654	Hodgkin lymphoma, lymphocyte depletion, diffuse fibrosis	Hodgkin lymphoma
9655	Hodgkin lymphoma, lymphocyte depletion, reticular	Hodgkin lymphoma
9659	Hodgkin lymphoma, nodular lymphocyte predominance	Hodgkin lymphoma
9661	Hodgkin granuloma	Hodgkin lymphoma
9662	Hodgkin sarcoma	Hodgkin lymphoma
9663	Hodgkin lymphoma, nodular sclerosis, NOS	Hodgkin lymphoma
9664	Hodgkin lymphoma, nodular sclerosis, cellular phase	Hodgkin lymphoma
9665	Hodgkin lymphoma, nodular sclerosis, grade 1	Hodgkin lymphoma
9667	Hodgkin lymphoma, nodular sclerosis, grade 2	Hodgkin lymphoma
9670	Malignant lymphoma, small B lymphocytic, NOS	Mature B-cell lymphomas
9671	Malignant lymphoma, lymphoplasmacytic	Mature B-cell lymphomas
9673	Mantle cell lymphoma	Mature B-cell lymphomas
9675	Malignant lymphoma, mixed small and large cell, diffuse	Mature B-cell lymphomas
9678	Primary effusion lymphoma	Mature B-cell lymphomas
9679	Mediastinal large B-cell lymphoma (C38.3)	Mature B-cell lymphomas
9680	Malignant lymphoma, large B-cell, diffuse, NOS	Mature B-cell lymphomas
9684	Malignant lymphoma, large B-cell, diffuse, immunoblastic, NOS	Mature B-cell lymphomas
9687	Burkitt lymphoma, NOS	Mature B-cell lymphomas
9688	T-cell/histiocyte rich large B-cell lymphoma	Mature B-cell lymphomas
9689	Splenic marginal zone B-cell lymphoma (C42.2)	Mature B-cell lymphomas
9690	Follicular lymphoma, NOS	Mature B-cell lymphomas
9691	Follicular lymphoma, grade 2	Mature B-cell lymphomas

9695	Follicular lymphoma, grade 1	Mature B-cell lymphomas
9698	Follicular lymphoma, grade 3	Mature B-cell lymphomas
9699	Marginal zone B-cell lymphoma, NOS	Mature B-cell lymphomas
9700	Mycosis fungoides (C44._)	Mature T- and NK-cell lymphomas
9701	Sezary syndrome	Mature T- and NK-cell lymphomas
9702	Mature T-cell lymphoma, NOS	Mature T- and NK-cell lymphomas
9705	Angioimmunoblastic T-cell lymphoma	Mature T- and NK-cell lymphomas
9708	Subcutaneous panniculitis-like T-cell lymphoma	Mature T- and NK-cell lymphomas
9709	Cutaneous T-cell lymphoma, NOS (C44._)	Mature T- and NK-cell lymphomas
9712	Intravascular large B-cell lymphoma (C49.9)	Mature T- and NK-cell lymphomas
9714	Anaplastic large cell lymphoma, T cell and Null cell type	Mature T- and NK-cell lymphomas
9716	Hepatosplenic T-cell lymphoma	Mature T- and NK-cell lymphomas
9717	Intestinal T-cell lymphoma	Mature T- and NK-cell lymphomas
9718	Primary cutaneous CD30+ T-cell lymphoproliferative disorder (C44._)	Mature T- and NK-cell lymphomas
9719	NK/T-cell lymphoma, nasal and nasal-type	Mature T- and NK-cell lymphomas
9724	Systemic EBV positive T-cell lymphoproliferative disease of childhood	Precursor cell lymphoblastic lymphoma
9725	Hydroa vacciniforme-like lymphoma	Precursor cell lymphoblastic lymphoma
9726	Primary cutaneous gamma-delta T-cell lymphoma	Precursor cell lymphoblastic lymphoma
9727	Precursor cell lymphoblastic lymphoma, NOS	Precursor cell lymphoblastic lymphoma
9728	Precursor B-cell lymphoblastic lymphoma	Precursor cell lymphoblastic lymphoma
9729	Precursor T-cell lymphoblastic lymphoma	Precursor cell lymphoblastic lymphoma
9731	Plasmacytoma, NOS	Plasma cell tumours
9732	Multiple myeloma (C42.1)	Plasma cell tumours
9733	Plasma cell leukaemia (C42.1)	Plasma cell tumours
9734	Plasmacytoma, extramedullary	Plasma cell tumours
9735	Plasmablastic lymphoma	Plasma cell tumours
9737	ALK positive large B-cell lymphoma	Plasma cell tumours
9738	Large B-cell lymphoma arising in HHV8-associated multicentric Castleman disease	Plasma cell tumours

9740	Mast cell sarcoma	Mast cell tumours
9741	Malignant mastocytosis	Mast cell tumours
9742	Mast cell leukaemia (C42.1)	Mast cell tumours
9750	Malignant histiocytosis	Neoplasms of histiocytes and accessory lymphoid cells
9751	Langerhans cell histiocytosis, NOS	Neoplasms of histiocytes and accessory lymphoid cells
9755	Histiocytic sarcoma	Neoplasms of histiocytes and accessory lymphoid cells
9756	Langerhans cell sarcoma	Neoplasms of histiocytes and accessory lymphoid cells
9757	Interdigitating dendritic cell sarcoma	Neoplasms of histiocytes and accessory lymphoid cells
9758	Follicular dendritic cell sarcoma	Neoplasms of histiocytes and accessory lymphoid cells
9759	Fibroblastic reticular cell tumour	Neoplasms of histiocytes and accessory lymphoid cells
9760	Immunoproliferative disease, NOS	Immunoproliferative diseases
9761	Waldenstrom macroglobulinemia (C42.0)	Immunoproliferative diseases
9762	Heavy chain disease, NOS	Immunoproliferative diseases
9764	Immunoproliferative small intestinal disease (C17._)	Immunoproliferative diseases
9800	Leukaemia, NOS	Leukaemias, NOS
9801	Acute leukaemia, NOS	Leukaemias, NOS
9805	Acute biphenotypic leukaemia	Leukaemias, NOS
9806	Mixed phenotype acute leukaemia with BCR-ABL1	Leukaemias, NOS
9807	Mixed phenotype acute leukaemia with MLL rearranged	Leukaemias, NOS
9808	Mixed phenotype acute leukaemia, B/myeloid, NOS	Leukaemias, NOS
9809	Mixed phenotype acute leukaemia, T/myeloid, NOS	Leukaemias, NOS
9811	B lymphoblastic leukaemia/lymphoma, NOS	Lymphoid leukaemias
9812	B lymphoblastic leukaemia/lymphoma with BCR-ABL1	Lymphoid leukaemias
9813	B lymphoblastic leukaemia/lymphoma with MLL rearranged	Lymphoid leukaemias
9814	B lymphoblastic leukaemia/lymphoma with TEL-AML1	Lymphoid leukaemias
9815	B lymphoblastic leukaemia/lymphoma with hyperdiploidy	Lymphoid leukaemias
9816	B lymphoblastic leukaemia/lymphoma with hypodiploidy	Lymphoid leukaemias
9817	B lymphoblastic leukaemia/lymphoma with IL3-IGH	Lymphoid leukaemias

9818	B lymphoblastic leukaemia/lymphoma with E2A-PBX1	Lymphoid leukaemias
9820	Lymphoid leukaemia, NOS	Lymphoid leukaemias
9823	B-cell chronic lymphocytic leukaemia/small lymphocytic lymphoma	Lymphoid leukaemias
9826	Burkitt cell leukaemia	Lymphoid leukaemias
9827	Adult T-cell leukaemia/lymphoma (HTLV-1 positive)	Lymphoid leukaemias
9831	T-cell large granular lymphocytic leukaemia	Lymphoid leukaemias
9832	Prolymphocytic leukaemia, NOS	Lymphoid leukaemias
9833	Prolymphocytic leukaemia, B-cell type	Lymphoid leukaemias
9834	Prolymphocytic leukaemia, T-cell type	Lymphoid leukaemias
9835	Precursor cell lymphoblastic leukaemia, NOS	Lymphoid leukaemias
9836	Precursor B-cell lymphoblastic leukaemia	Lymphoid leukaemias
9837	Precursor T-cell lymphoblastic leukaemia	Lymphoid leukaemias
9840	Acute myeloid leukaemia, M6 type	Myeloid leukaemias
9860	Myeloid leukaemia, NOS	Myeloid leukaemias
9861	Acute myeloid leukaemia, NOS	Myeloid leukaemias
9863	Chronic myeloid leukaemia, NOS	Myeloid leukaemias
9865	Acute myeloid leukaemia with DEK-NUP214	Myeloid leukaemias
9866	Acute promyelocytic leukaemia	Myeloid leukaemias
9867	Acute myelomonocytic leukaemia	Myeloid leukaemias
9869	Acute myeloid leukaemia	Myeloid leukaemias
9870	Acute basophilic leukaemia	Myeloid leukaemias
9871	Acute myeloid leukaemia with abnormal marrow eosinophils	Myeloid leukaemias
9872	Acute myeloid leukaemia, minimal differentiation	Myeloid leukaemias
9873	Acute myeloid leukaemia without maturation	Myeloid leukaemias
9874	Acute myeloid leukaemia with maturation	Myeloid leukaemias
9875	Chronic myelogenous leukaemia, BCR/ABL positive	Myeloid leukaemias
9876	Atypical chronic myeloid leukaemia, BCR/ABL negative	Myeloid leukaemias
9891	Acute monocytic leukaemia	Myeloid leukaemias
9895	Acute myeloid leukaemia with myelodysplasia-related changes	Myeloid leukaemias

9896	Acute myeloid leukaemia t(8;21)(q22;q22)	Myeloid leukaemias
9897	Acute myeloid leukaemia, 11q23 abnormalities	Myeloid leukaemias
9898	Myeloid leukaemia associated with Down Syndrome	Myeloid leukaemias
9910	Acute megakaryoblastic leukaemia	Myeloid leukaemias
9911	Acute myeloid leukaemia (megakaryoblastic) t(1;22) (p13;q13) RBM15-MKL1	Myeloid leukaemias
9920	Therapy related myeloid neoplasm	Myeloid leukaemias
9930	Myeloid sarcoma	Myeloid leukaemias
9931	Acute panmyelosis with myelofibrosis (C42.1)	Myeloid leukaemias
9940	Hairy cell leukaemia (C42.1)	Myeloid leukaemias
9945	Chronic myelonomocytic leukaemia, NOS	Other leukaemias
9946	Juvenile myelomonocytic leukaemia	Other leukaemias
9948	Aggressive NK-Cell leukaemia	Other leuakemias
9950	Polycythemia vera	Chronic myeloproliferative disorders
9960	Myeloproliferative neoplasm, NOS	Chronic myeloproliferative disorders
9961	Primary myelofibrosis	Chronic myeloproliferative disorders
9962	Essential thrombocythemia	Chronic myeloproliferative disorders
9963	Chronic neutrophilic leukaemia	Chronic myeloproliferative disorders
9964	Chronic eosinophilic leukaemia, NOS	Chronic myeloproliferative disorders
9965	Myeloid and lymphoid neoplasms with PDGFRA rearrangement	Chronic myeloproliferative disorders
9966	Myeloid neoplasms with PDGFRB rearrangement	Chronic myeloproliferative disorders
9967	Myeloid and lymphoid neoplasms with FGFR1 abnormalities	Chronic myeloproliferative disorders
9971	Polymorphic post-transplant lymphoproliferative disorder	Other hematologic disorders
9975	Myeloproliferative neoplasm, unclassifiable	Other hematologic disorders
9980	Refractory anaemia	Myelodysplastic syndromes
9982	Refractory anaemia with sideroblasts	Myelodysplastic syndromes
9983	Refractory anaemia with excess blasts	Myelodysplastic syndromes
9984	Refractory anaemia with excess blasts in transformation	Myelodysplastic syndromes
9985	Refractory cytopenia with multilineage dysplasia	Myelodysplastic syndromes

9986	Myelodysplastic syndrome with 5q deletion (5q-) syndrome	Myelodysplastic syndromes
9987	Therapy-related myelodysplastic syndrome, NOS	Myelodysplastic syndromes
9989	Myelodysplastic syndrome, NOS	Myelodysplastic syndromes
9991	Refractory neutropenia	Myelodysplastic syndromes
9992	Refractory thrombocytopenia	Myelodysplastic syndromes
